# Efficient vitamin A production in *Lipomyces starkeyi* through metabolic engineering of the β-carotene and retinoid pathways

**DOI:** 10.1186/s12934-026-03020-y

**Published:** 2026-05-12

**Authors:** Akari Kinoshita, Rikako Sato, Hibiki Higuchi, Shunichi Kobayashi, Kento Koketsu, Taro Watanabe, Hiroaki Takaku

**Affiliations:** 1 Kirin Central Research Institute, Kirin Holdings Company, Limited, 2-26-1, Muraoka-Higashi, Fujisawa, Kanagawa 251-8555 Japan; 2 Department of Applied Life Sciences, Niigata University of Pharmacy and Medical and Life Sciences, 265-1 Higashijima, Akiha-Ku, Niigata, 956-8603 Japan

**Keywords:** *Lipomyces starkeyi*, Oleaginous yeast, Vitamin A, β-carotene

## Abstract

**Background:**

Vitamin A, an essential micronutrient, is critical for vision, immune function, and cellular differentiation. Traditional vitamin A production methods, primarily based on chemical synthesis, pose significant environmental challenges. Microbial fermentation offers a sustainable alternative, but microbial production of vitamin A has yet to match chemical synthesis in terms of yield and cost-effectiveness. Oleaginous yeasts, such as *Lipomyces starkeyi*, which can synthesize high levels of acetyl-CoA and lipids, represent promising platforms for producing high-value lipophilic compounds, such as vitamin A.

**Results:**

*L. starkeyi* was engineered for the first time to produce vitamin A by introducing key enzymes in the β-carotene and retinoid biosynthetic pathways. This approach included integrating genes encoding lycopene cyclase/phytoene synthase (*McCarRP*), phytoene desaturase (*McCarB*), and β-carotene 15,15′-dioxygenase (*MbBlh*). Further optimization of the mevalonate pathway enabled the production of over 600 mg/L vitamin A in a 3-L fed-batch fermentation. Fe^2^⁺ supplementation improved yield, while a two-phase culture system using dodecane and butylated hydroxytoluene enhanced vitamin A recovery, with over 90% recovered in the extracellular phase.

**Conclusions:**

This study establishes *L. starkeyi* as a promising host for sustainable vitamin A production. Although significant improvements in yield were achieved, further optimization of pathway regulation and fermentation conditions is needed to reach economically competitive levels. These findings provide a foundation for developing *L. starkeyi* as a platform for large-scale production of vitamin A and other valuable terpenoids.

**Supplementary Information:**

The online version contains supplementary material available at 10.1186/s12934-026-03020-y.

## Introduction

Vitamin A is an essential micronutrient required for various physiological functions, including visual processes, immune regulation, and cellular differentiation. Humans primarily obtain vitamin A through metabolic conversion of dietary carotenoids, known as provitamin A, which are enzymatically cleaved to yield retinoids, such as retinal and retinol [1, 2]. Due to the broad biological significance of vitamin A, this vitamin and its derivatives have expanding applications in animal nutrition, cosmeceuticals, and pharmaceuticals, driving considerable growth in global market demand. In particular, retinol is widely recognized for its anti-aging effects in cosmeceuticals and its antioxidative and anti-infective properties utilized in nutraceutical and medical products [3, 4].

At present, commercial production of vitamin A is primarily achieved through chemical synthesis using petroleum-derived feedstocks. Developed in the early twentieth century, these methods pose significant environmental challenges due to high energy consumption and the generation of chemically stable, poorly biodegradable byproducts [5]. As a sustainable alternative, microbial production has been explored to enable more environmentally friendly manufacturing of vitamin A in response to these challenges.

Microbial fermentation is conducted under mild conditions, using sustainable raw materials to minimize environmental impact. Since the 2010s, several studies have reported *Escherichia coli* and *Saccharomyces cerevisiae* as platforms for vitamin A production. These studies improved vitamin A yield by engineering the mevalonate (MVA) pathway to boost precursor supply and by introducing heterologous genes involved in retinoid biosynthesis [6, 7]. Additionally, screening for retinal reductases and optimizing coenzyme balance have been applied to further enhance vitamin A production [8, 9]. Despite advances in strain engineering and pathway optimization, microbial vitamin A production has not replaced chemical synthesis because it remains economically uncompetitive. Increasing productivity through host selection and metabolic regulation is a viable strategy to achieve cost competitiveness.

Selecting an optimal production host depends on the specific characteristics of the target product [10]. In recent years, oleaginous yeasts with high cytosolic acetyl-CoA synthesis capacity have attracted attention as promising hosts. As acetyl-CoA is a common precursor for lipid and vitamin A biosynthesis, oleaginous yeasts, which efficiently produce large amounts of acetyl-CoA for lipid synthesis, are expected to achieve higher vitamin A yields. *Yarrowia lipolytica*, an oleaginous yeast, has shown remarkable production capacities, with yields reaching 39.5 g/L β-carotene and 4.86 g/L retinol, exceeding those reported for *E. coli* and *S. cerevisiae* [11, 12]. High retinol titers in *Y. lipolytica* have typically been achieved under extended fed-batch cultivation conditions [12, 13]. However, the impact of long-term cultivation on morphology and process stability remains an important consideration for industrial implementation [11, 14–16].

*Lipomyces starkeyi*, an oleaginous yeast, has broad metabolic capabilities, producing lipids from glucose and diverse sugars, including xylose, arabinose, and cellobiose [17, 18]. Unlike other oleaginous yeasts, such as *Y. lipolytica* and *Rhodosporidium toruloides*, *L. starkeyi* secretes α-amylase and α-glucosidase, enabling direct utilization of starch-derived substrates [19]. This characteristic may be advantageous for industrial applications, although its relevance to vitamin A production was not directly evaluated in the present study. With a lipid titer of 88 g/L, *L. starkeyi* is presumed to have high acetyl-CoA production capacity [20]. Its lipid content exceeds 85% of its dry cell weight, surpassing that of *Y. lipolytica*, making it an exceptionally promising host for the production of lipophilic compounds [21–23]. No dimorphism has yet been reported in *L. starkeyi*, and its stable unicellular morphology may be advantageous for industrial cultivation. However, similar morphological issues in other oleaginous yeasts such as *Y. lipolytica* can also be mitigated through genetic engineering [24]. Furthermore, several genetic engineering tools, including systems for genetic transformation, efficient targeted gene disruption, and overexpression, have been successfully developed [25–28]. Using these tools, *L. starkeyi* has demonstrated the ability to produce terpenoids such as α-zingiberene and lycopene, indicating its potential as a host for terpenoid production [29, 30].

In this study, we report the first successful production of vitamin A in *L. starkeyi*, an oleaginous yeast, by introducing three key enzymes in the β-carotene and retinoid biosynthesis pathways: McCarRP (lycopene cyclase/phytoene synthase) and McCarB (phytoene desaturase) from *Mucor circinelloides*, and MbBlh (β-carotene dioxygenase) from the uncultured marine bacterium 66A03. Vitamin A production was further enhanced by optimizing the MVA pathway. Fed-batch fermentation of the engineered strain yielded over 600 mg/L vitamin A, demonstrating the potential of *L. starkeyi* as a platform for high-level, sustainable vitamin A production.

## Materials and methods

### Strains and media

The yeast strains used in this study are listed in Table 1. *L. starkeyi ∆lslig4*, a strain with enhanced homologous recombination efficiency, was used to construct vitamin A–producing strains. The *∆lslig4* strain was derived from *L. starkeyi* CBS1807 [27]. For *S. cerevisiae* modifications, the parental strain YPH499 (Stratagene, CA, USA) was used. *L. starkeyi* and *S. cerevisiae* strains were grown at 30°C in YPD medium (1% Bacto™ Yeast Extract (Thermo Fisher Scientific, Waltham, MA, USA), 2% Bacto™ Peptone (Thermo Fisher Scientific), and 2% glucose). For solid media, 2% Bacto™ Agar (Thermo Fisher Scientific) was added, with or without supplementation of 100 μg/mL of Geneticin™ (Thermo Fisher Scientific), 100 μg/mL hygromycin B (FUJIFILM Wako Pure Chemical, Japan), 30 μg/mL nourseothricin (FUJIFILM Wako Pure Chemical), and/or 50 μg/mL Zeocin™ (Invitrogen Corporation, CA, USA). *S. cerevisiae* strains were cultured at 30°C in synthetic defined (SD) medium containing 0.17% yeast nitrogen base without amino acids and ammonium sulfate (Thermo Fisher Scientific), 0.5% ammonium sulfate (Nacalai Tesque, Japan), 2% glucose, and 0.14% yeast synthetic drop-out supplement without uracil, histidine, leucine, and tryptophan (Sigma-Aldrich, MO, USA). When required, uracil (100 μg/mL), leucine (30 μg/mL), tryptophan (80 μg/mL), and histidine (100 μg/mL) (FUJIFILM Wako Pure Chemical) were added to the medium. For solid media, 2% Bacto™ Agar was added.

**Table 1:** Yeast strains used in this study

*Lipomyces starkeyi*			
Strain Name	Parent strain	Description	Source
*Δlslig4*	CBS1807	*Δlslig4*::P_TDH3_-*Sh ble*-T_TDH3_	Oguro et al. [27]
KHNU-1	Δ*lslig4*	CBS1807, *Δlslig4*::P_70486_-*McCarRP*-T_70486_-P_TDH3_-*McCarB*-T_TDH3_-P_ACT1_-*KanR*-T_ACT1_	This study
KHNU-2	KHNU-1	KHNU-1, *Δlstgl3*::P_TDH3_-*XdCrtE*-T_TDH3_-P_ACT1_-*hph*-T_ACT1_	This study
KHNU-3	KHNU-2	KHNU-2, 18S rDNA:: P_ACT1_-*sNAT1*-T_ACT1_-P_70486_-*MbBlh*-T_70486_	This study
KHNU-4	KHNU-2	KHNU-2, *Δlsku80*::P_70486_-*MbBlh*-T_70486_-P_TDH3_-*MbBlh*-T_TDH3_-P_ACT1_-*sNAT1*-T_ACT1_	This study
KHNU-5	KHNU-3	KHNU-3, *Δlstgl4*::P_TDH3_-*HMG1*-T_TDH3_-P_ACT1_-*Sh ble*-T_ACT1_	This study
KHNU-6	KHNU-3	KHNU-3, *Δlstgl4*::P_TDH3_-*tHMG1*-T_TDH3_-P_ACT1_-*Sh ble*-T_ACT1_	This study
KHNU-7	KHNU-3	KHNU-3, *Δlsku80*::P_TDH3_-*tHMG1*-T_TDH3_-P_70486_-*ERG10*-T_70486_-P_TDH3_-*ERG13*-T_TDH3_-P_ACT1_-*Sh ble*-T_ACT1_	This study
KHNU-8	KHNU-2	KHNU-2, 18S rDNA::P_70486_-*CrCrtW*-T_70486_-P_ACT1_-*sNAT1*-T_ACT1_	This study
KHNU-9	KHNU-2	KHNU-2, 18S rDNA::P_70486_-*HpCrtZ*-T_70486_-P_ACT1_-*sNAT1*-T_ACT1_	This study
KHNU-10	KHNU-2	KHNU-2, 18S rDNA::P_70486_-*EuCrtZ*-T_70486_-P_ACT1_-*sNAT1*-T_ACT1_	This study
KHNU-11	KHNU-2	KHNU-2, 18S rDNA::P_70486_- *BrevCrtZ*-T_70486_ -P_3900_-*PspCrtW*-T_3900_-P_ACT1_-*sNAT1*-T_ACT1_	This study
*Saccharomyces cerevisiae*			
Strain Name	Parent strain	Description	Source
YPH499		*MATa ura3-52 lys2-801_amber ade2-101_ochre trp1-Δ63 his3-Δ200 leu2-Δ1*	Stratagene
KHNU-S1	YPH499	YPH499, *Δhmg1*::P_TDH3_-*tHMG1*-T_TDH3_-P_PGK1_-*KanR*-T_PGK1_	This study
KHNU-S2	KHNU-S1	KHNU-S1, pYES2(LEU2)-*CsPDP*, pYES2(HIS3)-empty	This study
KHNU-S3	KHNU-S1	KHNU-S1, pYES2(LEU2)-*CsPDP*, pYES2(HIS3)-*LsGGS1*	This study
KHNU-S4	KHNU-S1	KHNU-S1, pYES2(LEU2)-*CsPDP*, pYES2(HIS3)-*XdCrtE*	This study

*E. coli* HST08 (Takara Bio, Japan) was used for gene cloning and plasmid amplification and cultured at 37°C in LB broth (1% tryptone (Thermo Fisher Scientific), 0.5% Bacto™ Yeast Extract, and 0.5% NaCl) supplemented with 100 mg/L ampicillin for selection.

### General molecular biology techniques

Polymerase chain reactions (PCRs) were performed with DNA polymerases KOD Fx Neo and KOD One (TOYOBO, Japan), following the manufacturer’s instructions. Genomic DNA was extracted using Gen Toru-Kun for yeast (Takara Bio), and plasmid DNA was extracted using a QIAprep Spin Miniprep Kit (QIAGEN, Germany). Restriction enzymes were purchased from Takara Bio.

### Plasmid and strain construction

Primers used for plasmid and strain construction are listed in Table S1. Heterologous genes, including *Blh* from uncultured marine bacterium 66A03 (*MbBlh*), *CarRP* and *CarB* from *M. circinelloides* (*McCarRP* and *McCarB*), and *CrtE* from *Xanthophyllomyces dendrorhous* (*XdCrtE*), were codon-optimized for *L. starkeyi* expression and synthesized by Eurofins Genomics (Japan) (Table S2). The *XdCrtE* and *CsPDP* genes from *Croton stellatopilosus*, both codon-optimized for *S. cerevisiae*, were synthesized by Eurofins Genomics (Table S2).

To construct the *S. cerevisiae* strain KHNU-S1, a genomic integration cassette, pBlue-P_TDH3_-tHMG1-T_TDH3_-P_PGK1_-KanR-T_PGK1_, was assembled as follows. First, *HMG1* 5′- and 3′-untranslated regions (UTRs) were amplified from *S. cerevisiae* YPH499 genomic DNA using the primer sets ScHMG1_us_F/cHMG1_us_R and ScHMG1_ds_F/ScHMG1_ds_R, respectively. The vector backbone was amplified from pBluescript II KS(+) with pBlue_F1/pBlue_R1 primers. These DNA fragments were ligated using In-Fusion® Snap Assembly Master Mix (Takara Bio) to generate pBlue-HMG1us-HMG1ds. Next, *PGK1* promoter and terminator were amplified from YPH499 genomic DNA using PGK1p_F/PGK1p_R and PGK1t_F/PGK1t_R primer sets, respectively. The *KanR* gene was amplified from the plasmid pGKNEO2 [31] using G418r_F1/G418r_R1 primers. The vector backbone was amplified from pBluescript II KS(+) with pBlue_F2/pBlue_R2 primers. The resulting DNA fragments were ligated using In-Fusion® Snap Assembly Master Mix to construct pBlue-lox71-PGK1p-KanR-PGK1t-lox66. Finally, the vector backbone was amplified from pBlue-HMG1us-HMG1ds using pBlue_HMG1_vec_F/pBlue_HMG1_vec_R primers, the *KanR* expression cassette was amplified from pBlue-lox71-PGK1p-KanR-PGK1t-lox66 using G418r_F2/G418r_R2 primers, and the *tHMG1* expression cassette was amplified from pARS308-ERG12-tHMG1-URA [32] using ScHMG1_F/ScHMG1_R primers. These DNA fragments were assembled with In-Fusion® Snap Assembly Master Mix to yield the final plasmid pBlue-P_TDH3_-tHMG1-T_TDH3_-P_PGK1_-KanR-T_PGK1_.

To construct the *S. cerevisiae* expression plasmids pYES2(LEU2)-*CsPDP*, pYES2(HIS3)-*LsGGS1*, and pYES2(HIS3)-*XdCrtE*, the marker gene *URA3* in the parental vector pYES2/CT was first replaced with *LEU2* or *HIS3* to generate pYES2(LEU2)-empty or pYES2(HIS3)-empty, respectively. The *LEU2* and *HIS3* DNA fragments were amplified from *S. cerevisiae* genomic DNA using primer sets LEU2_F/LEU2_R and HIS3_F/HIS3_R, respectively. The resulting PCR products were assembled with a pYES2/CT backbone fragment, amplified using primers pYES2CT_F/pYES2CT_R, by In-Fusion® Snap Assembly Master Mix to obtain pYES2(LEU2)-empty and pYES2(HIS3)-empty.

To construct pYES2(LEU2)-*CsPDP*, the codon-optimized *CsPDP* gene for *S. cerevisiae* (synthesized by Eurofins Genomics) was amplified using primers CsPDP_Sc opt_F/CsPDP_Sc opt_R and assembled with the pYES2(LEU2)-empty backbone amplified using pYESCT_F2/pYESCT_R2 primers by In-Fusion® Snap Assembly Master Mix. To construct pYES2(HIS3)-*LsGGS1* and pYES2(HIS3)-*XdCrtE*, *LsGGS1* cDNA was amplified from *L. starkeyi* using LsGGS1_Sc opt_F/LsGGS1_Sc opt_R primers, and the codon-optimized *XdCrtE* gene for *S. cerevisiae* (synthesized by Eurofins Genomics) was amplified using XdCrtE_Sc opt_F/XdCrtE_Sc opt_R primers. Each amplified gene fragment was assembled with the pYES2(HIS3)-empty backbone (amplified using primers pYESCT_F2/pYESCT_R2) using In-Fusion® Snap Assembly Master Mix, yielding pYES2(HIS3)-*LsGGS1* and pYES2(HIS3)-*XdCrtE*.

The *S. cerevisiae* strain KHNU-S1 was constructed by transforming cells with plasmid pBlue-P_TDH3_-tHMG1-T_TDH3_-P_PGK1_-KanR-T_PGK1_ after digestion with XhoI and subsequent purification. KHNU-S2 was generated by introducing pYES2(LEU2)-*CsPDP* and pYES2(HIS3)-empty vectors into KHNU-S1. In the same manner, KHNU-S3 and KHNU-S4 were obtained by co-transforming KHNU-S1 with pYES2(LEU2)-*CsPDP* together with pYES2(HIS3)-*LsGGS1* and pYES2(HIS3)-*XdCrtE*, respectively.

To construct plasmids for β-carotene production in *L. starkeyi* strains KHNU-1 and KHNU-2, we developed the expression cassettes pKS/lig4/McCarRP-McCarB and pKS/tgl3/XdCrtE. For pKS/lig4/McCarRP-McCarB, we amplified the following DNA fragments from *L. starkeyi* genomic DNA: the 5′ and 3′ UTRs of *LsLIG4* (primers: lig4_us_F/lig4_us_R and lig4_ds_F/lig4_ds_R), the 70486 promoter and terminator regions (primers: 70486p_F/70486p_R and 70486t_F/70486t_R), and the *TDH3* promoter and terminator regions (primers: TDH3p_F/TDH3p_R and TDH3t_F/TDH3t_R). For gene expression in *L. starkeyi*, the *TDH3* promoter and the 70486 promoter region were chosen. The *TDH3* promoter has been previously used for high-level gene expression in *L. starkeyi* [26], and the 70486 promoter was selected based on RNA-seq data [19], identifying it as constitutively highly expressed. The codon-optimized *McCarRP* and *McCarB* genes for *L. starkeyi* were synthesized by Eurofins Genomics and amplified using primers McCarRP_F/McCarRP_R and McCarB_F/McCarB_R. For the *KanR* expression unit, *KanR* was amplified from pKS-kanR-LsKU70 [27] using primers KanRFw/KanRRv, and the vector backbone was amplified from pKS-18S-PTDH3-SLA1 [33] using primers vector(TDH3)Fw/vector(TDH3)Rv. These fragments were ligated using NEBuilder HiFi DNA Assembly (NEB, Japan) to form pKS/18S/hph/KanR. To construct pKS/18S/KanR/KanR, KanR and vector backbone were amplified from pKS/18S/hph/KanR using primers KanR_F/KanR_R and ACT1_F/ACT1_R, respectively. Subsequently, *KanR* and vector backbone were amplified from pKS/18S/KanR/KanR using primers G418_F/G418_R and vector_F/vector_R, respectively. The final plasmid, pKS/lig4/McCarRP-McCarB, was assembled from these DNA fragments using In-Fusion® Snap Assembly Master Mix.

For pKS/tgl3/XdCrtE construction, the 5′ and 3′ UTRs of *LsTGL3* (primers: tgl3_us_F/tgl3_us_R and tgl3_ds_F/tgl3_ds_R) and the *TDH3* promoter and terminator regions (primers: TDH3p_F2/TDH3p_R2 and TDH3t_F2/TDH3t_R2) were amplified. The codon-optimized *XdCrtE* gene for *L. starkeyi* was synthesized by Eurofins Genomics and amplified using primers XdCrtE_F/XdCrtE_R. For the *hph* expression unit, *hph* was amplified from pKS-hph-LsKU80 [27] using primers hphFw/hphRv, and the vector backbone was amplified from pKS-18S-PTDH3-SLA1 [33] using primers vector(TDH3)Fw/vector(TDH3)Rv. These fragments were ligated with NEBuilder HiFi DNA Assembly (NEB) to form pKS/18S/hph/hph. Subsequently, hph and vector backbone were amplified from pKS/18S/hph/hph using primers hph_F/hph_R and vector_F2/vector_R2, respectively. The final plasmid, pKS/tgl3/XdCrtE, was assembled using In-Fusion® Snap Assembly Master Mix.

For vitamin A production in *L. starkeyi* strains KHNU-3 and KHNU-4, plasmids pKS/18S/MbBlh and pKS/ku80/MbBlh-MbBlh were constructed. The 70486 promoter and terminator regions (primers: 70486p_F2/70486p_R2 and 70486t_F2/70486t_R2) were amplified from *L. starkeyi* genomic DNA. The codon-optimized *MbBlh* gene was synthesized by Eurofins Genomics and amplified using primers MbBlh_F/MbBlh_R. For the *sNAT1* expression unit, *sNAT1* was amplified from pKS-sNAT1-LsURA3 [27] using primers natFw/natRv, and the vector backbone was amplified from pKS-18S-PTDH3-SLA1 [33] using primers vector(TDH3)Fw/vector(TDH3)Rv. These fragments were ligated with NEBuilder HiFi DNA Assembly (NEB) to form pKS/18S/hph/sNAT1. To construct pKS/18S/sNAT1/sNAT1, sNAT1 and vector backbone were amplified from pKS/18S/hph/sNAT1 using primers sNAT1_F2/sNAT1_R2 and ACT1_F/ACT1_R, respectively. Vector backbone containing *sNAT1* was amplified from pKS/18S/sNAT1/sNAT1 using primers vec_18S_sNAT1_F/vec_18S_sNAT1_R. The final plasmid, pKS/18S/MbBlh, was assembled using In-Fusion® Snap Assembly Master Mix.

For pKS/ku80/MbBlh-MbBlh, the 5′ and 3′ UTRs of *LsKU80* (primers: KU80_us_F/KU80_us_R and KU80_ds_F/KU80_ds_R) and the *TDH3* promoter and terminator regions (primers: TDH3p_F3/TDH3p_R3 and TDH3t_F3/TDH3t_R3) were amplified from genomic DNA. The first *MbBlh* expression cassette was amplified from pKS/18S/MbBlh (primers: 70486p_F3/70486t_R3), and the second *MbBlh* gene was amplified from the codon-optimized *MbBlh* gene (primers: MbBlh_F2/MbBlh_R2). The *sNAT1* expression unit and vector backbone were amplified from pKS/18S/sNAT1/sNAT1 using primers vector_F3/vector_R3 and sNAT1_F/sNAT1_R. These fragments were ligated using In-Fusion® Snap Assembly Master Mix to complete the construction of pKS/ku80/MbBlh-MbBlh.

For plasmids pKS/tgl4/HMG1, pKS/tgl4/tHMG1, and pKS/ku80/ERG10-ERG13-tHMG1, the 5′ and 3′ UTRs of *LsTGL4* and *TDH3* promoter and terminator regions were amplified (primers: tgl4_us_F/tgl4_us_R, tgl4_ds_F/tgl4_ds_R, TDH3p_F4/TDH3p_R4, and TDH3t_F4/TDH3t_R4) from *L. starkeyi* genomic DNA. The *Sh ble* expression unit was constructed by amplifying *Sh ble* from pKS-Sh ble-LsLIG4 [27] using primers Sh bleFw/Sh bleRv, and the vector backbone was amplified from pKS-18S-PTDH3-SLA1 [33] using primers vector(TDH3)Fw/vector(TDH3)Rv. These fragments were ligated with NEBuilder HiFi DNA Assembly (NEB) to form pKS/18S/hph/Sh ble. To construct pKS/18S/Sh ble/Sh ble, *Sh ble* and vector backbone were amplified from pKS/18S/hph/Sh ble using primers ble_F2/ble_R2 and ACT1_F/ACT1_R, respectively, and then amplified from pKS/18S/Sh ble/Sh ble using primers ble_F/ble_R and vector_F4/vector_R4. The final plasmid, pKS/tgl4/HMG1, was completed by ligating the *HMG1* gene (primers: HMG1_F/HMG1_R) amplified from *L. starkeyi* cDNA. For pKS/tgl4/tHMG1, the *tHMG1* gene was amplified from *L. starkeyi* cDNA using primers tHMG1_F/tHMG1_R, and the *TDH3* promoter was amplified from genomic DNA using primers TDH3p_F4/TDH3p_R5. All remaining steps were identical to those used for the construction of pKS/tgl4/HMG1, with *HMG1* replaced by *tHMG1*.

For pKS/ku80/ERG10-ERG13-tHMG1, a preliminary plasmid, pKS/lig4/ERG10-ERG13-HMG1(ble), was first generated. The 5′ and 3′ UTRs of the *LsLIG4* gene, the 70486 promoter and terminator regions, and the *TDH3* promoter and terminator regions were amplified from *L. starkeyi* genomic DNA using primers LsLIG4_5′UTR_2k_Fw/LsLIG4_5′UTR_Rv, LsLIG4_3′UTR_2k_Fw/LsLIG4_3′UTR_2k_Rv, 70486p(ERG10)Fw/70486p(ERG10)Rv, 70486t(ERG10)Fw/70486t(ERG10)Rv, TDH3p(ERG13)Fw/TDH3p(ERG13)Rv, TDH3t(ERG13)Fw/TDH3t(ERG13)Rv, 70486p(HMG1)Fw/70486p(HMG1)Rv, and 70486t(HMG1)Fw/70486t(HMG1)Rv. The *ERG10*, *ERG13*, and *HMG1* genes were amplified from *L. starkeyi* cDNA using primers ERG10Fw/ERG10Rv, ERG13Fw/ERG13Rv, and HMG1Fw/HMG1Rv, respectively. The *sNAT1* unit was amplified from pKS-18S-PTDH3-TYR1 [33] using primers P_sNAT1_T_Fw/P_sNAT1_T_Rv, and the vector backbone was amplified from pKS-18S-hph [26] using primers VectorFw/VectorRv. Fragments were assembled using the NEBuilder HiFi DNA Assembly kit (NEB) to generate pKS/lig4/ERG10-ERG13-HMG1(sNAT1). The *sNAT1* cassette was replaced with *Sh ble* by joining fragments from pKS/lig4/ERG10-ERG13-HMG1(sNAT1) (primers: ACT1_F/ACT1_R) and pKS/18S/hph/Sh ble (primers: ble_F2/ble_R2) using In-Fusion® Snap Assembly Master Mix, yielding pKS/lig4/ERG10-ERG13-HMG1(ble).

For the construction of pKS/ku80/ERG10-ERG13-tHMG1, the 5′ and 3′ UTRs of *LsKU80* were amplified from *L. starkeyi* genomic DNA (primers: ku80_us_F/ku80_us_R2 and ku80_ds_F/ku80_ds_R). The *tHMG1* cassette was amplified from pKS/tgl4/tHMG1 (primers: tHMG1_F2/tHMG1_R2), and the *ERG10*/*ERG13*/*Sh ble* cassette was amplified from pKS/lig4/ERG10-ERG13-HMG1(ble) (primers: 70486p_F/ACT1t_R). The vector backbone was amplified from pKS/18S/hph/Sh ble (primers: vector_F4/vector_R4). The five fragments were assembled using In-Fusion® Snap Assembly Master Mix to generate pKS/ku80/ERG10-ERG13-tHMG1.

For the construction of *L. starkeyi* strains KHNU-1 to KHNU-7, plasmids were introduced as follows. KHNU-1 was generated by transforming the ∆*lslig4* strain with NotI-digested pKS/lig4/McCarRP-McCarB, containing the *McCarRP* and *McCarB* genes from *M. circinelloides* for β-carotene biosynthesis. The KHNU-2 strain was then created by transforming KHNU-1 with NotI-digested pKS/tgl3/XdCrtE, carrying the *XdCrtE* gene from *X. dendrorhous* for further carotenoid biosynthesis. The KHNU-3 and KHNU-4 strains were constructed by transforming KHNU-2 with ApaI-digested pKS/18S/MbBlh and NotI-digested pKS/ku80/MbBlh-MbBlh, respectively, to express β-carotene 15,15′-dioxygenase (*MbBlh*) from uncultured marine bacterium 66A03. Finally, KHNU-5, KHNU-6, and KHNU-7 strains were constructed by transforming KHNU-3 with NotI-digested pKS/tgl4/HMG1, pKS/tgl4/tHMG1, and pKS/ku80/ERG10-ERG13-tHMG1, respectively, to enhance the MVA pathway for improved terpenoid production.

### Yeast transformation

*S. cerevisiae* transformation was performed as follows. *S. cerevisiae* cells were precultured overnight in 4 mL of YPD medium. Then, 2% of the preculture was inoculated into 50 mL of YPD and cultured till mid-logarithmic phase. Cells were harvested by centrifugation (2260 × g, 5 min), and the supernatant was discarded. Subsequent steps were performed on ice. The cell pellet was washed with 20 mL of sterile distilled water and centrifuged (2260 × g, 4°C, 5 min). Then, the pellet was washed twice with 50 mL of 1 M sorbitol. After centrifugation (2260 × g, 4°C, 5 min), the cells were suspended in 200 μL of 1 M sorbitol. Then, 40 μL of this suspension was mixed with > 1 μg of transforming DNA and electroporated using 0.2 cm cuvettes under the following conditions: 25 μF capacitance, 7.5 kV/cm electric field strength, and 200 Ω resistance. Electroporated cells were resuspended in 1 mL of 1 M sorbitol and centrifuged (2260 × g, 4°C, 5 min). After the supernatant was removed, the remaining suspension was plated onto SD medium lacking leucine, histidine, or both.

*L. starkeyi* transformation was performed as previously described [28]. Cells were cultured overnight in 50 mL of YPD medium till mid-logarithmic phase. Briefly, *L. starkeyi* cells were harvested by centrifugation (4000 × g, 4°C, 5 min), and the supernatant was discarded. The pellet was resuspended in 10 mL of buffer A (1 mM Tris–HCl, 0.1 mM EDTA, 0.2 M lithium acetate, pH 8.0) and incubated at 30°C with gentle shaking for 45 min. Subsequently, 100 μL of 1 M dithiothreitol was added, followed by incubation for 15 min under the same conditions. Cells were washed twice with 50 mL of ice-cold sterile distilled water and once with 3 mL of 0.5 M ice-cold sucrose. After centrifugation (4000 × g, 4°C, 5 min), 40 μL of the cell suspension was mixed with 1 μg of transforming DNA, incubated on ice for 5 min, and electroporated using 0.2 cm cuvettes under the following conditions: 25 μF capacitance, 3.75 kV/cm electric field strength, and 800 Ω resistance. Electroporated cells were resuspended in 1 mL of ice-cold 0.5 M sucrose, transferred to 5 mL of YPD medium supplemented with 0.5 M sucrose, and incubated overnight at 30°C with gentle shaking. After recovery, the culture was centrifuged (4000 × g, RT, 5 min), and the pellet was resuspended in sterile distilled water and plated onto selective medium.

### Culture conditions

*S. cerevisiae* was precultured in YPD medium at 30°C and 300 rpm overnight. Then, 2% precultures were inoculated into 4 mL of fresh YPD medium in large test tubes and cultured for 72 h. At mid-log phase, 2% galactose was added to induce the expression of geranylgeranyl diphosphate (GGPP) synthase and GGPP phosphatase for GGOH production. Then, 800 μL dodecane containing 1% (w/v) butylated hydroxytoluene (BHT) was added for in situ extraction of GGOH.

*L. starkeyi* was precultured in YPD medium at 30°C and 300 rpm for 2 days. The precultures were inoculated into 4 mL of fresh YPD medium in large test tubes to an initial OD600 of approximately 0.2 and cultured for 96 h. At mid-log phase, 800 μL of dodecane containing 1% (w/v) BHT was added for in situ vitamin A extraction.

### Analysis of geranylgeraniol (GGOH)

In the GGPP synthase assay, GGOH production was quantified as follows. The dodecane layer from the cultures of GGOH-producing strains was collected and directly analyzed by gas chromatography/mass spectrometry (Shimadzu, Japan) equipped with an SH-I-5Sil MS column (30 m × 0.25 mm, 0.25 μm, Shimadzu). Samples were injected in split mode (1:10). The oven temperature was maintained at 100°C for 2 min, increased to 300°C at a rate of 20°C/min, and held for 10 min. Electron ionization was performed at 70 eV, with the ion source and transfer line temperatures set to 200°C and 250°C, respectively. Mass spectra were recorded over a range of 20–600 m/z, and GGOH was identified by characteristic fragment ions at 69, 81, and 93 m/z.

### Analysis of carotenoids and vitamin A

Intracellular carotenoids and retinoids were extracted as follows. Yeast cells were harvested (5000 × g, RT, 5 min) from 500 µL of culture, resuspended in 500 µL of ethyl acetate, containing 1 g of 0.5 mm glass beads and disrupted using a Multi-Beads Shocker (Yasui Kikai, Japan) at 2500 rpm for 15 min at 4°C. The disrupted mixtures were centrifuged, and the supernatants were analyzed. For extracellular retinoids in biphasic cultures with dodecane, cultures were centrifuged, and the upper dodecane phase was directly analyzed by high-performance liquid chromatography (HPLC). Retinoid (retinal and retinol) concentrations in the dodecane phase were calculated based on the volume of the aqueous phase. Quantification was performed using an HPLC LC-20 AD (Shimadzu) equipped with a C18 column (Inertsil ODS-2, 5 µm, 250 × 4.6 mm, GL Sciences Inc., Japan) and a photodiode array detector. The column oven was set to 40°C. The mobile phase comprised ethyl acetate (solvent A) and 90% (v/v) methanol in water (solvent B). The HPLC gradient was as follows: 0–16 min, linear increase from 0 to 60% A; 16–23 min, held at 60% A; 23–28 min, increased to 100% A and held; 28–33 min, decreased to 0% A and held for column equilibration. Carotenoids and retinoids were detected at 450 nm and 350 nm, respectively.

### Copy number estimation with quantitative real-time PCR

Whole-genome DNA was isolated using the DNeasy UltraClean Microbial Kit (QIAGEN). Copy numbers of integrated genes were determined using quantitative real-time PCR (qRT-PCR) using SYBR Premix Ex Taq II (Tli RNaseH Plus) (Takara Bio) on a Thermal Cycler Dice® Real Time System IV (Takara Bio). Each 25 µL reaction contained 12.5 μL of SYBR Premix Ex Taq II, 0.2 μL of each primer (50 μM), 1 μL of genomic DNA, and 11.1 μL of sterile water. Thermal cycling conditions were as follows: 95°C for 30 s (initial denaturation), followed by 40 cycles of 95°C for 5 s and 60°C for 30 s. Dissociation analysis was performed after incubating at 95°C for 15 s, 60°C for 30 s, and 95°C for 15 s. All assays were conducted in technical triplicate. Primer sequences are listed in Table S3. The *ACT1* gene was used as an internal reference. The relative copy number of *MbBlh* in each strain was calculated relative to the two-copy *MbBlh* strain using the 2^−ΔΔCT^ method.

### Gene expression analysis with qRT-PCR

Total RNA was extracted from *L. starkeyi* cells using the NucleoSpin® RNA Plant and Fungi kit (Takara Bio). First-strand cDNA was synthesized using PrimeScript RT reagent kits (Takara Bio) according to the manufacturer’s instructions. Gene expression was analyzed by qRT-PCR using SYBR Premix Ex Taq II (Tli RNaseH Plus) (Takara Bio) on the Thermal Cycler Dice® Real Time System IV (Takara Bio). Reaction mixture (volume: 25 µL) contained 12.5 μL SYBR Premix Ex Taq II, 0.2 μL of each primer (50 μM), 1 μL cDNA, and 11.1 μL sterile water. The thermal cycle conditions were as follows: 95°C for 30 s, followed by 40 cycles at 95°C for 5 s and 60°C for 30 s. Dissociation analysis was performed at 95°C for 15 s, 60°C for 30 s, and 95°C for 15 s. qRT-PCRs were performed in biological triplicate. Primer sequences are listed in Table S3. *ACT1* was used as an internal control, and relative gene expression was calculated using the 2^−ΔCT^ method.

### Fed-batch fermentation

For fed-batch fermentation, engineered yeast cells were precultured in 250 mL of YPD medium for 2 days, and 100 mL of the culture was inoculated into 1 L of YPD medium with 0.7 mM FeSO_4_·7H_2_O in a 3-L bioreactor. The pH was maintained at 5.5 using pH–stat feeding with 4 N ammonia solution. Fermentation was conducted at 30°C, with 1.5 vvm aeration and 600 rpm agitation, while dissolved oxygen was maintained above 3.5 ppm. The fed-batch phase started 24 h after inoculation with feeding of 50% (w/v) glucose. For two-phase fermentation, 200 mL of dodecane containing 1% (w/v) BHT was added 24 h after inoculation. OD600 was measured using UV 1900i (Shimadzu).

### Measurement of triacylglycerol (TAG) content

Cells were harvested from 1 mL of liquid culture by centrifugation (5000 × g, RT, 1 min), washed once with 1 mL of phosphate-buffered saline (PBS), and resuspended in 1 mL of PBS. The optical density at 600 nm of the cell suspension was adjusted to approximately 10. After centrifugation (5000 × g, RT, 1 min), the resultant cell pellet was incubated at 70 °C for 5 min, and then stored at − 20 °C for 5min. Subsequently, 500 μL of PBS and 1 g of 0.5 mm glass beads were added, and the cells were disrupted at room temperature using a Multi-Beads Shocker (Yasui Kikai) for 30 cycles (30 s agitation followed by 30 s rest) at 2500 rpm. After cell disruption, an additional 500 μL of PBS was added, and the mixture was incubated at room temperature with vigorous rotation using a Micro Mixer (EYELA, Japan). The total TAG content in the homogenized cell suspension was enzymatically analyzed using a LabAssay™ Triglyceride (FUJIFILM Wako Pure Chemical) according to the manufacturer’s instructions.

### Statistical analysis

Statistical analysis was performed using Student’s t-test or Dunnett’s test in Python (version 3.11.8) with statsmodels (version 0.13.5) and SciPy (version 1.14.1). Differences were considered significant at P < 0.05 after Holm adjustment for multiple comparisons.

## Results

### Construction of the β-carotene biosynthetic pathway in *L. starkeyi*

**Fig. 1:**
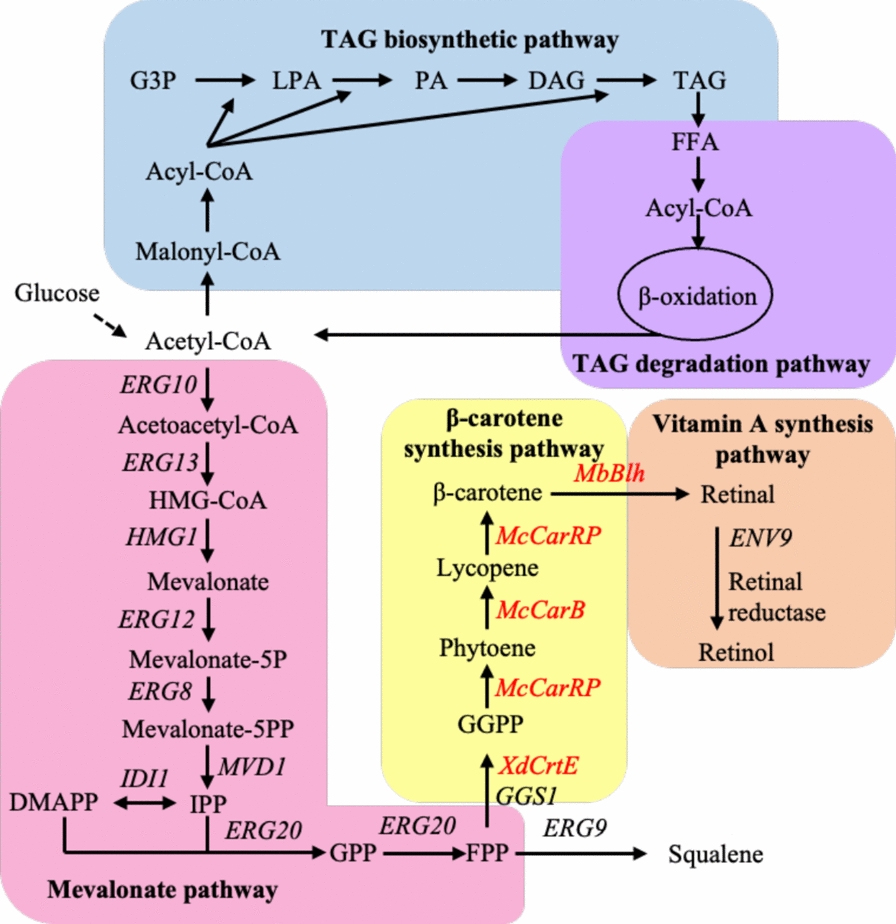
Overview of metabolic pathways engineered for vitamin A biosynthesis in *Lipomyces starkeyi*. Schematic representation of the metabolic network linking the MVA pathway, β-carotene synthesis, vitamin A (retinoid) synthesis, and TAG metabolism in *L. starkeyi*. The MVA pathway (pink) produces terpenoid precursors DMAPP and IPP, condensed into GPP, FPP, and GGPP. The β-carotene pathway (yellow) converts GGPP to β-carotene via phytoene synthase/lycopene cyclase (*McCarRP*) and phytoene desaturase (*McCarB*) from *Mucor circinelloides*. Heterologous GGPP synthase (*XdCrtE*) from *Xanthophyllomyces dendrorhous* enhances flux from FPP to GGPP. The vitamin A pathway (orange) converts β-carotene to retinal via β-carotene dioxygenase (*MbBlh*) from marine bacterium 66A03, followed by endogenous reduction to retinol by retinal reductase (ENV9) or other retinal reductases. TAG biosynthesis (blue) and degradation (purple) provide storage and acetyl-CoA recycling to support terpenoid metabolism. Heterologous genes are shown in red; endogenous genes of *L. starkeyi* are shown in black. *MVA* mevalonate, *TAG* triacylglycerol, *DMAPP* dimethylallyl diphosphate, *IPP* isopentenyl diphosphate, *GPP* geranyl diphosphate, *FPP* farnesyl diphosphate, *GGPP* geranylgeranyl diphosphate, *FFA* free fatty acid, *G3P* glycerol-3-phosphate, *HMG-CoA* 3-hydroxy-3-methylglutaryl-CoA, *LPA* lysophosphatidic acid, *PA* phosphatidic acid, *DAG* diacylglycerol

Figure 1 shows the MVA pathway, the β-carotene biosynthesis pathway, and the retinol and retinal (hereafter referred to as vitamin A) biosynthesis pathway in *L. starkeyi*. MVA pathway–related genes and the GGPP synthase gene in the β-carotene biosynthesis pathway were identified in *L. starkeyi* via BLASTP in NCBI using *S. cerevisiae* gene sequences as queries (Table S4). *L. starkeyi* does not naturally produce carotenoids. The *L. starkeyi* ∆*lslig4* strain, constructed by inserting a zeocin resistance gene into the *LsLIG4* locus, exhibits growth and lipid production levels comparable to those of the wild-type strain CBS1807 [27].

**Fig. 2:**
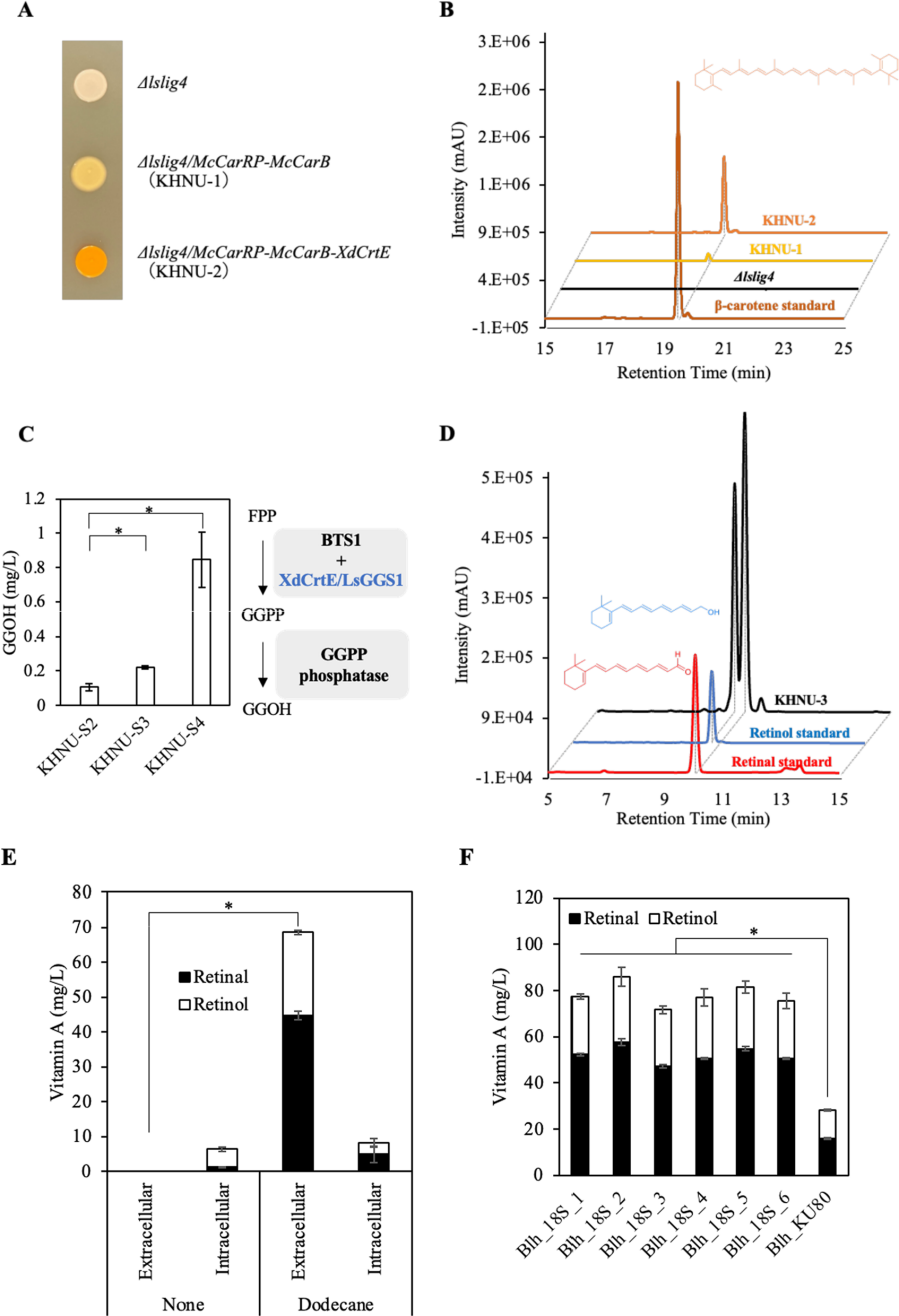

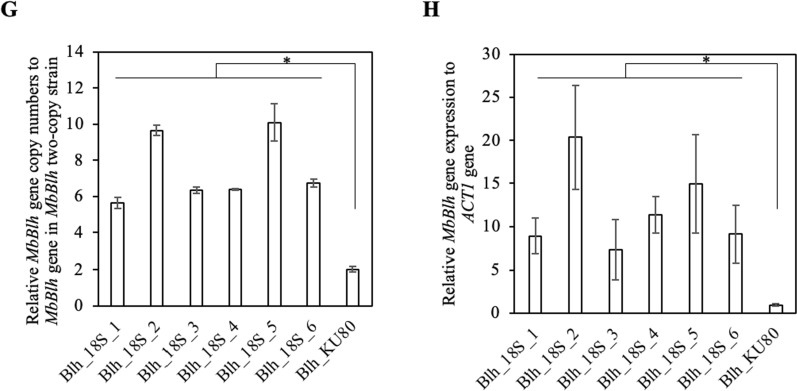
Construction of β-carotene and vitamin A biosynthetic pathways in *Lipomyces starkeyi* and evaluation of *MbBlh* copy number effects on vitamin A production. **A** Phenotypic comparison of *L. starkeyi Δlslig4* (control), KHNU-1, and KHNU-2 strains showing progressive pigmentation with the introduction of heterologous β-carotene genes (*McCarB*, *McCarRP*, *XdCrtE*). **B** HPLC chromatograms of β-carotene extracted from KHNU-1 and KHNU-2, compared with an authentic standard. **C** Evaluation of GGPP synthase activity at 72 h in *S. cerevisiae* strains KHNU-S2 (endogenous *BTS1*), KHNU-S3 (overexpressing *L. starkeyi GGS1*), and KHNU-S4 (overexpressing *XdCrtE*). GGOH quantified by GC/MS; inset shows the GGPP phosphatase assay schematic. **D** HPLC chromatograms confirming the production of retinal and retinol in *L. starkeyi* KHNU-3 (Blh_18S_1) expressing *MbBlh* from marine bacterium 66A03, with peaks identified against authentic standards. **E** Vitamin A production by KHNU-3 (Blh_18S_1) at 72 h under single-phase and two-phase culture; dodecane (20%, v/v) with 1% (w/v) BHT was used for in situ extraction. Retinal (filled bars) and retinol (open bars) are shown separately in stacked bar graphs. **F** Vitamin A titers at 96 h among KHNU-3 (Blh_18S_1–6) and KHNU-4 (Blh_KU80) strains with different *MbBlh* copy numbers. Retinal (filled bars) and retinol (open bars) are presented separately. **G** Quantitative PCR analysis of *MbBlh* gene copy numbers in KHNU-3 (Blh_18S_1–6) strains. **H** Relative *MbBlh* transcript levels in KHNU-3 (Blh_18S_1–6) strains determined by qRT-PCR after 48 h. Data represent mean ± SEM of three independent experiments; asterisks indicate statistically significant differences (Student’s t test or Dunnett’s test; * P < 0.05). *HPLC* high-performance liquid chromatography, *GGOH* geranylgeraniol, *GC/MS* gas chromatography/mass spectrometry, *GGPP* geranylgeranyl diphosphate, *BHT* butylated hydroxytoluene, *PCR* polymerase chain reaction, *qRT-PCR* quantitative real-time PCR, *SEM* standard error of mean

To produce the retinal precursor, β-carotene, in *L. starkeyi*, genes encoding lycopene cyclase/phytoene synthase (*McCarRP*) and phytoene desaturase (*McCarB*) from *M. circinelloides*, under high-expression promoters, were inserted at the zeocin locus in the ∆*lslig4* strain to construct strain KHNU-1 with a heterologous β-carotene biosynthesis pathway (Fig. S1A). The ∆*lslig4* strain showed white cells, whereas KHNU-1 cells were slightly yellow (Fig. 2A). In *S. cerevisiae*, enhanced genetic modifications in the β-carotene pathway increase β-carotene synthesis and change cell color from yellow to orange [34]. Although KHNU-1 synthesizes β-carotene, its production is low, indicating that further pathway enhancement is needed (Fig. 2B).

GGPP synthase is one of the key enzymes for β-carotene production. In *S. cerevisiae* and *Y. lipolytica*, introducing the GGPP synthase gene into strains overexpressing lycopene cyclase/phytoene synthase and phytoene desaturase enhances β-carotene synthesis, suggesting weak endogenous GGPP synthase activity [34, 35]. Since the activity of endogenous GGPP synthase may be weak in *L. starkeyi*, as in *S. cerevisiae* and *Y. lipolytica*, we investigated *L. starkeyi* GGPP synthase activity using a geranylgeraniol (GGOH)-producing *S. cerevisiae* strain (KHNU-S2). Because intracellular GGPP is chemically unstable and rapidly metabolized, it is difficult to quantify directly. Therefore, geranylgeraniol (GGOH), a dephosphorylated derivative of GGPP, is commonly used as an indirect reporter of GGPP synthesis in yeast isoprenoid engineering [36]. In this system, KHNU-S2 exhibits weak endogenous GGPP synthase (*BTS1*) activity and expresses a heterologous GGPP phosphatase from *C. stellatopilosus*, enabling sensitive detection of GGOH [37]. Because the same GGPP phosphatase is expressed under identical conditions in all strains and its activity has been reported not to be rate-limiting for GGOH formation [38], differences in GGOH accumulation primarily reflect differences in GGPP synthase activity.

The KHNU-S3 strain, which overexpresses *L. starkeyi GGS1* (a homolog of *S. cerevisiae BTS1*) in the KHNU-S2 background, showed only a slight increase in GGOH production, indicating low GGPP synthase activity (Fig. 2C). It has been reported that in the yeast *S. cerevisiae* and *Y. lipolytica*, overexpression of the gene encoding GGPP synthase (*XdCrtE*) from *X. dendrorhous* enabled more efficient β-carotene production [11, 34]. As expected, the KHNU-S4 strain, obtained by overexpressing the *XdCrtE* gene in the KHNU-S2 strain, showed markedly increased GGOH production (Fig. 2C), demonstrating that XdCrtE efficiently enhances β-carotene biosynthesis in *L. starkeyi*.

Accordingly, KHNU-2 was constructed by integrating *XdCrtE* into the *LsTGL3* locus of KHNU-1 for overexpression (Fig. S1B). Deletion of *LsTGL3*, which was chosen as the integration site, did not affect cell growth or lipid accumulation, as the Δ*lstgl3* strain exhibited phenotypes comparable to those of the control strain Δ*lslig4* (Fig. S2). Compared with KHNU-1, KHNU-2 showed significantly higher β-carotene production and deeper orange coloration (Fig. 2A and B). These results indicate that *XdCrtE* integration into KHNU-1 enhanced the metabolic flux from farnesyl pyrophosphate (FPP) to GGPP, improving conversion efficiency.

### Construction of vitamin A–producing *L. starkeyi* strains

β-carotene 15,15′-dioxygenase is a key enzyme in the vitamin A biosynthetic pathway, catalyzing the central cleavage of β-carotene to form retinal, a direct precursor of retinol [39]. Previous studies have shown that introducing a β-carotene dioxygenase gene from the uncultured marine bacterium 66A03 (MbBlh) into *S. cerevisiae* markedly enhanced the conversion of β-carotene to vitamin A [7, 9]. Moreover, multi-copy integration of *MbBlh* increased vitamin A titers in *S. cerevisiae* and *Y. lipolytica* [9, 12]. Based on these findings, the KHNU-3 strain was constructed by integrating *MbBlh* into the 18S rDNA locus of the β-carotene–producing strain KHNU-2 (Fig. S1C). The resulting KHNU-3 (Blh_18S_1) strain produced both retinal and retinol, which were individually identified and quantified by HPLC using authentic standards, without introducing an exogenous retinal reductase. These results indicate that endogenous reductase activity in *L. starkeyi* is sufficient to convert at least part of the produced retinal to retinol (Fig. 2D). The 18S rDNA integration approach has previously been applied to construct multi-copy expression strains in *L. starkeyi* [26]. Accordingly, vitamin A production in KHNU-3 strains could vary depending on the *MbBlh* copy number at the 18S rDNA locus. To test this hypothesis, six KHNU-3 strains, KHNU-3 (Blh_18S_1) to KHNU-3 (Blh_18S_6), were randomly selected and analyzed for vitamin A production and *MbBlh* copy number.

Vitamin A in KHNU-3 (Blh_18S_1) was detected only intracellularly (6 mg/L) and not in the culture supernatant (Fig. 2E). Two-phase cultivation systems with hydrophobic extractants such as dodecane have been widely used for culturing vitamin A–producing microorganisms to relieve intracellular storage limitations and protect vitamin A from oxidative degradation [6, 7]. Since *L. starkeyi* exhibited similar growth in media with or without 20% (v/v) dodecane (Fig. S3), a two-phase culture system was applied to KHNU-3 (Blh_18S_1) using dodecane supplemented with 1% (w/v) BHT to prevent vitamin A oxidation. This approach enabled the production of 76.7 mg/L vitamin A (69 mg/L extracellular and 7.7 mg/L intracellular) (Fig. 2E), demonstrating that dodecane and BHT effectively enhance vitamin A production in *L. starkeyi*. This method was used in subsequent experiments.

To evaluate the effects of *MbBlh* copy number on vitamin A production, the KHNU-4 strain was constructed by integrating two copies of *MbBlh* into the *LsKU80* locus of KHNU-2 (Fig. S1D). Vitamin A production was then compared among KHNU-4 and KHNU-3 (Blh_18S_1–6) strains under two-phase cultivation. KHNU-4 produced ~ 30 mg/L vitamin A, whereas all KHNU-3 (Blh_18S_1–6) strains exceeded 70 mg/L (Fig. 2F). In contrast, β-carotene titer in KHNU-4 reached ~ 40 mg/L, while in KHNU-3 (Blh_18S_1–6) strains, it was ~ 10 mg/L (Fig. S4), indicating that MbBlh-mediated conversion of β-carotene to retinal was significantly enhanced in the KHNU-3 derivatives.

Quantitative PCR analysis revealed that KHNU-3 (Blh_18S_1), KHNU-3 (Blh_18S_3), KHNU-3 (Blh_18S_4), and KHNU-3 (Blh_18S_6) contained six copies of *MbBlh*, whereas KHNU-3 (Blh_18S_2) and KHNU-3 (Blh_18S_5) harbored ten copies (Fig. 2G). Despite this difference, no significant differences in the production of vitamin A or β-carotene were observed between the six- and ten-copy strains. Gene expression analysis further showed that *MbBlh* transcript levels were relatively higher in the ten-copy strains than in the six-copy strains (Fig. 2H). The high transcription levels observed in KHNU-3 strains may reflect not only increased gene copy number but also the strong transcriptional activity of the 18S rDNA locus used for integration. In contrast, KHNU-4 carries *MbBlh* integrated at the *LsKU80* locus, which may exhibit different transcriptional characteristics. However, the lack of proportional improvement in vitamin A yield with increased *MbBlh* expression suggests that other steps downstream or upstream of retinal formation may have become rate-limiting.

To avoid promoter titration effects in subsequent metabolic engineering aimed at enhancing vitamin A biosynthesis, KHNU-3 (Blh_18S_1), one of the six-copy strains, was selected as the representative KHNU-3 strain for further analyses.

### Metabolic engineering of the MVA pathway to enhance vitamin A production

**Fig. 3:**
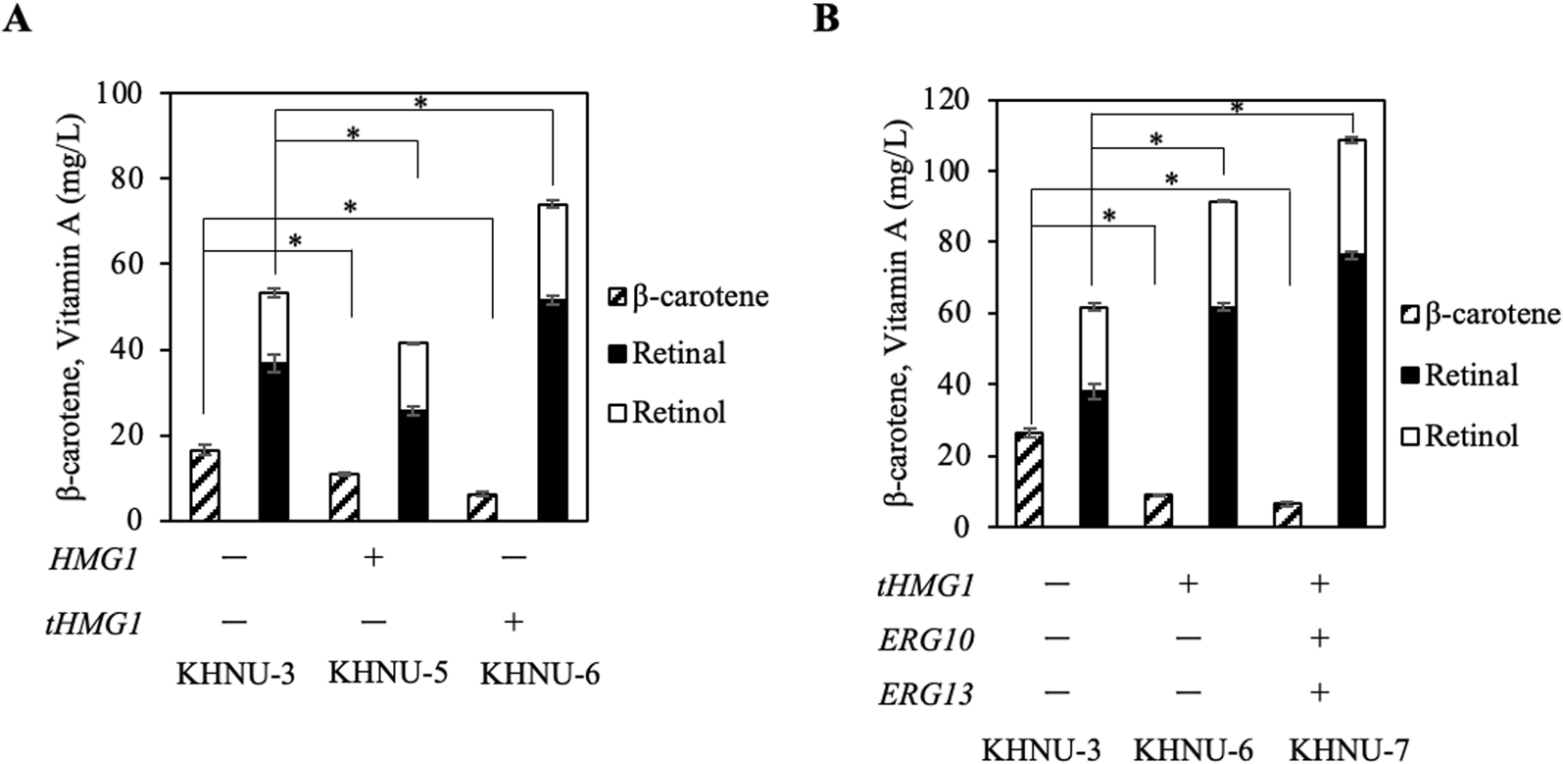
Enhancement of vitamin A production in *Lipomyces starkeyi* through reinforcement of the MVA pathway. **A** Effects of overexpression of *HMG1* and truncated *HMG1* (*tHMG1*) on vitamin A production after 96 h of cultivation. The KHNU-3 strain was used as the parental control. Retinal (filled bars) and retinol (open bars) are shown separately. β-carotene is indicated by hatched bars. **B** Further enhancement of vitamin A production after 96 h of cultivation by co-overexpression of *tHMG1*, *ERG10* (acetyl-CoA acetyltransferase), and *ERG13* (HMG-CoA synthase). Retinal (filled bars), retinol (open bars), and β-carotene (hatched bars) are presented. Data shown in Fig. 3A and Fig. 3B were obtained from independent cultivation experiments conducted under identical conditions. Data represent mean ± SEM from three independent experiments. An asterisk indicates significant differences (Dunnett’s test; * P < 0.05). *MVA* mevalonate, *SEM* standard error of mean

The MVA pathway provides essential precursors for vitamin A biosynthesis (Fig. 1). The rate-limiting step in this pathway is the reduction of HMG-CoA to MVA, catalyzed by HMG-CoA reductase (HMGR). Previous studies have shown that overexpression of one or more MVA pathway genes enhances terpenoid production in the oleaginous yeasts *Y. lipolytica* and *L. starkeyi* [14, 24, 30, 40]. A primary strategy to enhance vitamin A biosynthesis is to increase flux through the MVA pathway via metabolic engineering. Initially, we constructed the KHNU-5 strain by integrating the *L. starkeyi HMG1* gene, which encodes HMGR, into the *LsTGL4* locus of KHNU-3 to achieve overexpression (Fig. S1E). However, KHNU-5 showed no improvement in the production of β-carotene or vitamin A (Fig. 3A). We also confirmed that the *LsTGL4* deletion strain, and the double-deletion strain lacking *LsTGL3* and *LsTGL4*, which were target sites for gene integration, exhibited growth and lipid accumulation comparable to the ∆*lslig4* strain, consistent with the ∆*lstgl3* phenotype of the ∆*lstgl3* strain (Fig. S2, Table S5). In mammals, HMGR inactivation via feedback regulation of sterol synthesis is mediated by the enzyme’s N-terminal transmembrane domain [41]. N-terminally truncated HMGR forms have been shown to enhance terpenoid biosynthesis in yeasts by circumventing feedback-induced proteolysis, thereby improving cytoplasmic stability of the enzyme [42]. Accordingly, we constructed the KHNU-6 strain by introducing and overexpressing the truncated *L. starkeyi tHMG1* gene, encoding tHmg1p lacking the N-terminal transmembrane domain (residues 1–529), into KHNU-3 (Fig. S1E). KHNU-6 produced more vitamin A than KHNU-5 (Fig. 3A), suggesting that feedback regulation via Hmg1p degradation in response to sterols exists in *L. starkeyi*, and that tHmg1p enhanced MVA pathway flux, increasing vitamin A synthesis.

We then constructed KHNU-7 by inserting and overexpressing *L. starkeyi tHMG1*, *ERG10* (acetyl-CoA acetyltransferase), and *ERG13* (HMG-CoA synthase) at the *LsKU80* locus of KHNU-3 (Fig. S1F). Compared with KHNU-6, KHNU-7 showed further enhancement in vitamin A production, which might be attributed to reinforcement of the MVA pathway via *ERG10* and *ERG13* overexpression (Fig. 3B). The strains analyzed in Fig. 3A and B were evaluated in separate cultivation experiments performed during different stages of strain construction under identical conditions. Therefore, absolute titers between panels are not directly comparable, whereas the relative trends within each experiment remain consistent.

### Effects of culture conditions on vitamin A production

**Fig. 4:**
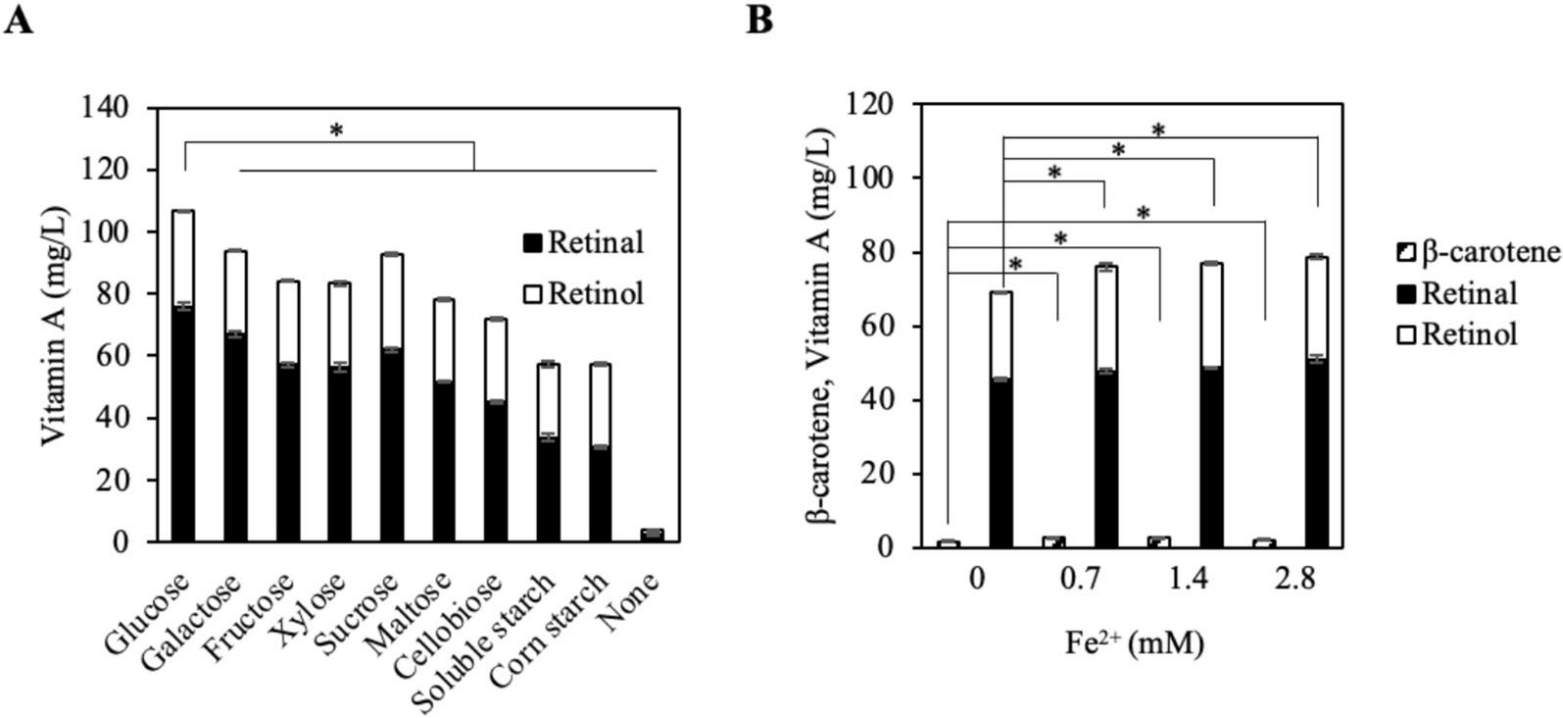
Effects of carbon source and Fe^2^⁺ supplementation on vitamin A production in *Lipomyces starkeyi* KHNU-7. **A** Influence of different carbon sources on vitamin A production by engineered *L. starkeyi* KHNU-7. Cells were cultivated in YP medium supplemented with 2% (w/v) of each indicated carbon source for 96 h under two-phase culture with 20% (v/v) dodecane containing 1% (w/v) BHT. Retinal (filled bars) and retinol (open bars) are shown separately. **B** Effects of Fe^2^⁺ supplementation on vitamin A production after 96 h. KHNU-7 was cultured in YPD medium with varying FeSO₄ concentrations (0–2.8 mM). Retinal (filled bars), retinol (open bars), and β-carotene (hatched bars) are presented. Data represent mean ± SEM of three independent experiments. Asterisks indicate statistically significant differences (Dunnett’s test; * P < 0.05). *BHT* butylated hydroxytoluene, *SEM* standard error of mean

*L. starkeyi* can utilize a wide range of sugars, including glucose, fructose, xylose, sucrose, cellobiose, and starch, as carbon sources [17, 43]. To assess the effects of carbon source on vitamin A production, the KHNU-7 strain was cultivated separately with each sugar. All tested substrates supported vitamin A biosynthesis, with glucose yielding the highest vitamin A titer (Fig. 4A), indicating it is the most favorable carbon source.

MbBlh activity is positively regulated by Fe^2^⁺ and inhibited by the iron-chelating agent phenanthroline [44]. Supplementing Fe^2^⁺ increased vitamin A and β-carotene levels in KHNU-7 (Fig. 4B). A clear increase was observed at 0.7 mM Fe^2^⁺, whereas higher concentrations (1.4 and 2.8 mM) provided minimal or no additional improvement, indicating that carotenoid metabolism responds positively to Fe^2^⁺ but plateaus beyond 0.7 mM Fe^2^⁺.

### Large-scale production of vitamin A in fed-batch fermentation

**Fig. 5:**
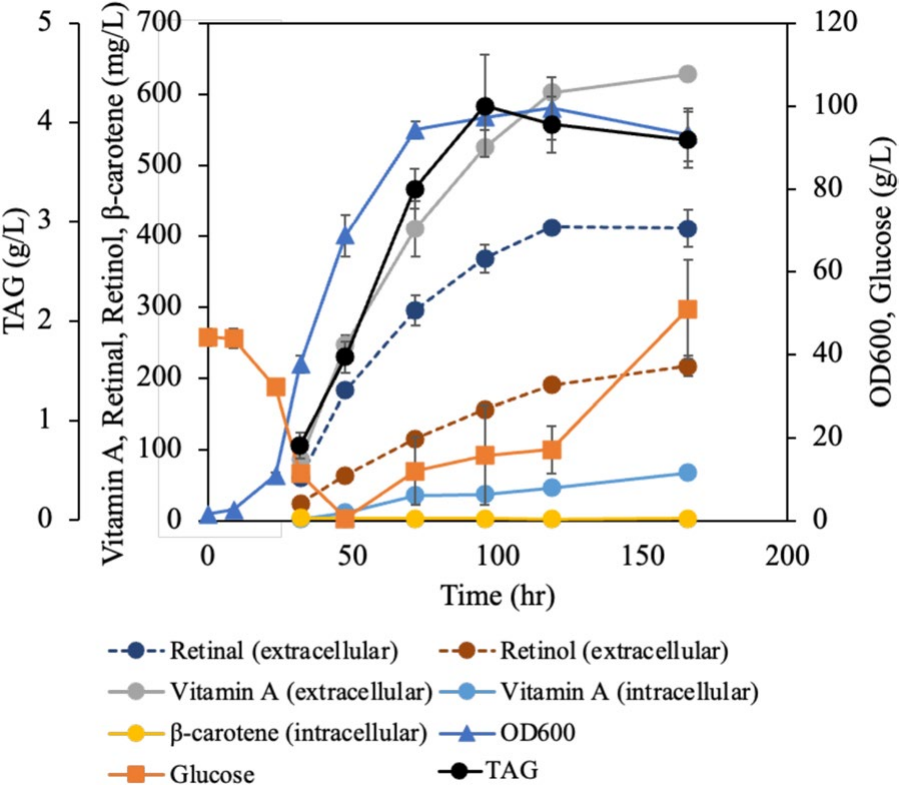
Fed-batch fermentation of the engineered *Lipomyces starkeyi* KHNU-7 strain for vitamin A production. Time-course profiles of cell growth (OD600), extracellular and intracellular vitamin A, intracellular β-carotene, glucose concentration, and intracellular triacylglycerol (TAG) during 168 h of cultivation. Extracellular retinal and retinol are additionally shown as separate dashed lines to distinguish their dynamic changes during fermentation. Data represent the mean ± SEM from three independent experiments. *TAG* triacylglycerol, *SEM* standard error of mean

To evaluate the vitamin A production capacity of the engineered strain KHNU-7, fed-batch fermentation was performed in YPD medium supplemented with Fe^2+^ and 20% dodecane containing 1% BHT using a 3-L bioreactor. After 160 h, KHNU-7 produced 627 mg/L extracellular and 67 mg/L intracellular vitamin A (Fig. 5). In the 3-L bioreactor, over 90% of total vitamin A was recovered in the extracellular dodecane phase, consistent with test tube–based batch cultures. β-carotene accumulation was minimal, suggesting its conversion to retinal is not a rate-limiting step. The main vitamin A production period (32–119 h) largely overlapped with that of triacylglycerol (TAG) (32–96 h) (Fig. 5), indicating that both pathways are activated in parallel and share acetyl-CoA as a metabolic substrate in *L. starkeyi*. Accordingly, TAG-hyperproducing strains with improved cytosolic acetyl-CoA supply may serve as effective hosts for TAG synthesis and high-level vitamin A production.

## Discussion

Microbial biosynthesis of vitamin A and related carotenoids offers a sustainable alternative to petrochemical synthesis, driven by advances in yeast metabolic engineering and process optimization. In this study, *L. starkeyi* was established as a novel host for vitamin A biosynthesis, achieving 627 mg/L extracellular vitamin A in fed-batch two-phase fermentation. Although this titer is lower than the 5.21 g/L and 5.4 g/L achieved with *S. cerevisiae* [9] and *Y. lipolytica* [13], respectively, *L. starkeyi* can serve as an alternative oleaginous yeast platform for vitamin A biosynthesis and provide a basis for further metabolic and process optimization.

### Comparison with the established yeast hosts

*S. cerevisiae* is widely used for retinoid biosynthesis due to its mature genetic toolkit. Shi et al. [9] achieved a record 5.21 g/L retinol titer via *MbBlh* and *SsBCO* co-expression, NADPH regeneration (POS5Δ17), and multi-copy gene integration. Limited cytosolic acetyl-CoA pool and lack of lipid storage constrain terpenoid precursor supply, while intracellular retinol accumulation can cause oxidative and membrane stress, requiring careful process control and product recovery strategies [8, 45]. In this study, a biphasic culture system using a dodecane overlay was employed to facilitate vitamin A recovery and to reduce oxidative degradation of retinoids. Therefore, the use of biphasic extraction should not be interpreted as a physiological limitation of *L. starkeyi*. Instead, it represents a practical process strategy to enhance product recovery and stability during fermentation. Nevertheless, the implementation of organic overlay systems at industrial scale may require additional engineering considerations, such as solvent handling and phase separation efficiency.

In contrast, *Y. lipolytica*, an oleaginous yeast with high acetyl-CoA flux, has reached retinol titers of 5.4 g/L via co-expression of *MbBlh* and *RDH12* under optimized fed-batch conditions [13]. However, dimorphic growth and morphological shifts under stress hinder aeration, mixing, and process control in large-scale bioreactors [14, 15]. These studies highlight that while both yeasts can achieve high titers, physiological and morphological constraints limit their industrial robustness.

High retinol titers in *Y. lipolytica* (4.86–5.4 g/L) have been achieved under fed-batch cultivation for approximately 104–168 h [12, 13]. In the present study, *L. starkeyi* required a comparable fermentation duration (approximately 168 h; Fig. 5) to reach maximum vitamin A titers. Therefore, the value of *L. starkeyi* does not lie in reduced cultivation time, but potentially in its stable unicellular morphology under prolonged production conditions, which may facilitate oxygen transfer and process control during scale-up. We note, however, that comparable morphological stability can also be engineered in other hosts such as *Y. lipolytica*.

### Advantages of *L. starkeyi* as a vitamin A production host

Our findings demonstrate that *L. starkeyi* is a non-dimorphic oleaginous yeast capable of vitamin A biosynthesis while maintaining stable unicellular morphology under the conditions tested in this study. Its high lipid accumulation (> 85% of dry cell weight) may provide intracellular storage capacity for hydrophobic metabolites. In addition, the strong endogenous supply of cytosolic acetyl-CoA associated with lipid metabolism may be beneficial for terpenoid biosynthesis. However, these potential advantages were not directly compared experimentally with other hosts in the present study.

*L. starkeyi* also exhibits broad substrate versatility, utilizing carbohydrates such as starch, xylose, and cellobiose [17]. Starch degradation is facilitated by secreted α-amylase and α-glucosidase [19], indicating the potential to use starch-based feedstocks. However, this property was cited here as a general physiological feature of the host based on previous studies and was not directly evaluated for vitamin A production in the present work. Therefore, the practical relevance of starch utilization for retinoid production by *L. starkeyi* remains to be examined in future studies. Thus, *L. starkeyi* combines robust metabolic capacity, morphological simplicity, and feedstock flexibility, making it an attractive host for sustainable retinoid biomanufacturing.

In addition to its physiological characteristics, *L. starkeyi* may also offer practical advantages as a relatively underexplored microbial production host. Compared with more extensively engineered yeasts such as *S. cerevisiae* and *Y. lipolytica*, the use of *L. starkeyi* may provide greater flexibility for future strain development and commercialization. Such practical considerations may increase the value of developing *L. starkeyi* as an alternative host for retinoid and terpenoid production.

### Effects of Fe^2^⁺ supplementation on retinoid biosynthesis

A key finding of this study is the pronounced effects of Fe^2^⁺ on vitamin A synthesis. Supplementation with 0.7 mM Fe^2^⁺ increased vitamin A titers and reduced β-carotene accumulation, consistent with Fe^2^⁺ acting as a cofactor for β-carotene 15,15′-dioxygenase (*MbBlh*) [44]. This enhancement likely results from improved enzyme activity through cofactor stabilization. Concentrations above 0.7 mM did not further increase titers, which might be attributed to the oxidative stress caused by Fe^2^⁺-catalyzed radical formation. *L. starkeyi* maintained robust growth under Fe^2^⁺ supplementation, suggesting that its lipid reserves and antioxidant systems mitigate reactive oxygen species, maintaining enzymatic stability and cellular redox homeostasis. These findings indicate that optimizing trace metal availability is an effective complementary strategy to enhance retinoid productivity in *L. starkeyi*.

### Expansion of *L. starkeyi* as a platform for carotenoid and terpenoid production

Beyond vitamin A, *L. starkeyi* can synthesize diverse high-value carotenoids when specific heterologous oxygenase and ketolase genes are introduced into the β-carotene-producing strain KHNU-2 (Fig. S5A). KHNU-8, expressing β-carotene ketolase (CrtW) from *Chlamydomonas reinhardtii*, produced canthaxanthin. KHNU-9, containing β-carotene hydroxylase (CrtZ) from *Haematococcus pluvialis*, generated β-cryptoxanthin (Fig. S5B). Expression of *CrtZ* from *Erwinia uredovora* in KHNU-10 facilitated zeaxanthin biosynthesis, while co-expression of *CrtZ* from *Brevundimonas* sp. SD212 and *CrtW* from *Paracoccus* sp. N81106 in KHNU-11 enabled the conversion of β-carotene to astaxanthin (Fig. S5B). Quantitative analysis revealed production levels of 3.4 mg/L canthaxanthin in KHNU-8, 11.7 mg/L β-cryptoxanthin in KHNU-9, 1.1 mg/L zeaxanthin in KHNU-10, and 13.0 mg/L astaxanthin in KHNU-11 under the tested conditions (Fig. S5C).

These results demonstrate that *L. starkeyi* efficiently supports diverse oxidative conversions in the carotenoid pathway, highlighting its potential as a versatile platform for the biosynthesis of oxygenated carotenoids.

This broad product scope positions *L. starkeyi* as a versatile platform for the biosynthesis of lipophilic terpenoids, including vitamin A and nutraceutical pigments, such as astaxanthin and β-cryptoxanthin. Compared with *S. cerevisiae* and *Y. lipolytica*, *L. starkeyi* forms large intracellular lipid droplets that may serve as storage compartments for hydrophobic metabolites. Such intracellular lipid storage could potentially reduce product toxicity and oxidative degradation, although the stabilizing effect of lipid droplets on retinoids was not directly evaluated in this study. This feature may therefore represent a potential advantage of oleaginous yeasts for the production of highly hydrophobic terpenoids. Thus, *L. starkeyi* may represent a promising oleaginous yeast chassis for carotenoid and terpenoid production.

### Metabolic implications and industrial outlook

Metabolic engineering of the MVA pathway via overexpression of *tHMG1*, *ERG10*, and *ERG13* significantly improved vitamin A titer, consistent with other yeast systems [9, 12, 45]. The lack of further titer increase with additional *MbBlh* copies suggests that precursor or cofactor availability, rather than dioxygenase activity, limits biosynthetic flux under these conditions. Concurrent accumulation of TAG and retinoids indicates a cooperative relationship between lipid synthesis and retinoid formation, wherein TAG serves as both a storage compartment and a redox buffer for hydrophobic intermediates [12, 46]. In addition, the ability of *L. starkeyi* to tolerate biphasic solvent extraction with dodecane/BHT demonstrates its robustness and facilitates downstream recovery.

Although current titers in *L. starkeyi* have not yet reached those of optimized *S. cerevisiae* and *Y. lipolytica* systems, its high lipid storage capacity, broad substrate utilization, and tolerance to oxidative and solvent stress offer potential industrial advantages. Integration of NADPH regeneration, iron homeostasis optimization, and dynamic TAG mobilization, combined with fermentation strategies such as carbon-to-nitrogen ratio control and oxygen regulation, may further enhance retinol production to levels comparable to those achieved in high-performing microbial systems. In addition, *L. starkeyi*’s ability to synthesize multiple carotenoids underscores its potential as a versatile platform for terpenoid production. At the same time, several limitations of *L. starkeyi* as a production host should be recognized. In particular, vitamin A titers remain lower than those reported for the best engineered *S. cerevisiae* and *Y. lipolytica* strains, and the full potential of alternative feedstocks and redox engineering was not explored in this study. Therefore, further optimization of pathway regulation, precursor supply, cofactor balance, and fermentation strategy will be necessary to establish *L. starkeyi* as a competitive industrial host.

## Conclusions

This study demonstrates that *L. starkeyi* is a robust, adaptable, and industrially relevant platform for retinoid and terpenoid biosynthesis. Compared with conventional microbial hosts, it offers morphological stability, high acetyl-CoA flux, substantial lipid storage, broad substrate utilization, and strong tolerance to hydrophobic and oxidative stresses. Successful production of canthaxanthin, β-cryptoxanthin, zeaxanthin, and astaxanthin shows that *L. starkeyi* can produce not only vitamin A but also a range of valuable carotenoids. These features highlight *L. starkeyi* as a promising next-generation oleaginous yeast platform for sustainable, industrial-scale production of vitamin A and related terpenoids from renewable feedstocks.

## Supplementary Information

Additional file 1.

## Data Availability

All data generated or analyzed during this study are included in this published article and its Additional files.

## Abbreviations

BHT: Butylated hydroxytoluene
GGOH: Geranylgeraniol
GGPP: Geranylgeranyl diphosphate
HPLC: High-performance liquid chromatography
MVA: Mevalonate
PCR: Polymerase chain reaction
qRT-PCR: Quantitative real-time PCR
SD: Synthetic defined
TAG: Triacylglycerol
UTR: Untranslated region
