# Supplementary material for: Efficient vitamin A production in Lipomyces starkeyi through metabolic engineering of the β-carotene and retinoid pathways
Source: Microb Cell Fact. 2026 May 12;25:153. doi: 10.1186/s12934-026-03020-y (PMC13344046; doi:10.1186/s12934-026-03020-y)
Supplement: Supplementary file 1 — Additional file 1. [file 12934_2026_3020_MOESM1_ESM.docx]

**Additional file**

**Supplementary Material**

**Efficient Vitamin A Production in *Lipomyces starkeyi* through Metabolic Engineering of the β-Carotene and Retinoid Pathways**

Akari Kinoshita^1^, Rikako Sato^2^, Hibiki Higuchi^1^, Shunichi Kobayashi^1^, Kento Koketsu^1^, Taro Watanabe^1^, and Hiroaki Takaku^2,*^

^1^ Kirin Central Research Institute, Kirin Holdings Company, Limited, 2-26-1, Muraoka-Higashi, Fujisawa 251-8555, Kanagawa, Japan

^2^ Department of Applied Life Sciences, Niigata University of Pharmacy and Medical and Life Sciences, 265-1 Higashijima, Akiha-ku, Niigata 956-8603, Japan

*Corresponding author: Hiroaki Takaku,

E-mail address: htakaku@nupals.ac.jp

Phone: +81 250 25 5119; Fax: +81 250 25 5021

**Supplementary Tables**

**Table S1 Primers used for plasmid and strain construction in this study**

Overlapping sequences for In-Fusion Snap Assembly are shown in lowercase, while template-specific annealing regions are shown in uppercase.

| Primer name | Primer sequence (5′-3′) |
| --- | --- |
| lig4_us_F | ggcggagcctatggaaagcggccgcTCGACGTATAACATACAAATAGCCCAAC |
| lig4_us_R | gtagagtagagcgagAGTTACCACAATTATATGCACATGGAG |
| lig4_ds_F | caactctatactcctGCCTGTTATAGAAGTCAAGTTCGC |
| lig4_ds_R | caaataggggttccgcggcggccgcGCGAGGATCATACATATCCTCACAACTC |
| vector_F | AGGATATGTATGATCCTCGCgcggccgccgcggaacccct |
| vector_R | TTGTATGTTATACGTCGAgcggccgctttccatagg |
| G418_F | ttccaccatcaccacccgctacatTTACCGTCTACCGCTGACGTTTTG |
| G418_R | AGGAGTATAGAGTTGAATTTAATGGACG |
| McCarRP_F | ttactatctatattcaactttcacaATGTTGTTGACGTATATGGAAGTCCAC |
| McCarRP_R | ggttcgagaggaagcgagacgacgtTTATATCGTATTCAGATTCCGCATTTTGC |
| McCarB_F | tatcttactctaatccacattcgcaATGTCCAAAAAGCACATAGTCATCATTG |
| McCarB_R | agatagaagaccatcaaccgcacacTTAAATGACATTCGAATTATGGACCC |
| 70486p_F | CTCGCTCTACTCTACTCTAGTCTAACTCTAGTTTAC |
| 70486p_R | ggacttccatatacgtcaacaacatTGTGAAAGTTGAATATAGATAGTAAGAGCTTTTTCG |
| TDH3p_F | ggcaagcccaccctgctcttcccTTAATTTGCTGAAGCGGTTTGCC |
| TDH3p_R | tgatgactatgtgctttttggacatTGCGAATGTGGATTAGAGTAAGATAGATAAC |
| 70486t_F | aatgcggaatctgaatacgatataaACGTCGTCTCGCTTCCTCTCGAACC |
| 70486t_R | accgcttcagcaaattaagGGAAGAGCAGGGTGGGC |
| TDH3t_F | ggtccataattcgaatgtcatttaaGTGTGCGGTTGATGGTCTTCTATCTTC |
| TDH3t_R | aacgtcagcggtagacggtaaATGTAGCGGGTGGTGATGGTG |
| tgl3_us_F | agcctatggaaagcggccgcGACGTCCAGCATATGCCCTG |
| tgl3_us_R | TTGTTCTAATACTTGTGCTTCTGGGC |
| tgl3_ds_F | GACCTTGAAGGGGTAGGGAGTG |
| tgl3_ds_R | aggggttccgcggcggccgcGTTGGACGCGTCGGTTGATG |
| vector_F2 | atcaaccgacgcgtccaacGCGGCCGCCGCGGAACCCC |
| vector_R2 | gggcatatgctggacgtcGCGGCCGCtttccatagg |
| hph_F | caccacccgctacatTTACCGTCTACCGCTGACG |
| hph_R | taccccttcaaggtcAGGAGTATAGAGTTGAATTTAATGGACGTTG |
| XdCrtE_F | tatcttactctaatccacattcgcaATGGACTATGCCAACATACTTACCG |
| XdCrtE_R | agatagaagaccatcaaccgcacacTTATAACGGGATATCAGCGAGCTTTTTC |
| TDH3p_F2 | caagtattagaacaaTTAATTTGCTGAAGCGGTTTGC |
| TDH3p_R2 | cggtaagtatgttggcatagtccatTGCGAATGTGGATTAGAGTAAGATAG |
| TDH3t_F2 | aagctcgctgatatcccgttataaGTGTGCGGTTGATGGTCTTCTATC |
| TDH3t_R2 | ATGTAGCGGGTGGTGATGGTG |
| vec_18S_sNAT1_F | caccctgctcttcccAACTAGCTCAAGGGACGTGCTATTC |
| vec_18S_sNAT1_R | gagtagagcgagAGGAGTATAGAGTTGAATTTAATGGACG |
| MbBlh_F | ttactatctatattcaactttcacaATGGGCCTTATGCTGATTGATTG |
| MbBlh_R | ggttcgagaggaagcgagacgacgtTTAGTTTTTGATCTTGATCCGACTAGAATG |
| 70486p_F2 | cctCTCGCTCTACTCTACTCTAGTCTAACTCTAGTTTAC |
| 70486p_R2 | accaatcaatcagcataaggcccatTGTGAAAGTTGAATATAGATAGTAAGAGCTTTTTCG |
| 70486t_F2 | tagtcggatcaagatcaaaaactaaACGTCGTCTCGCTTCCTCTCGAACC |
| 70486t_R2 | GGGAAGAGCAGGGTGGGCTTG |
| KU80_us_F | ggcggagcctatggaaagcggccgcGCTCTCCGACCTAGGTGTTCATGGG |
| KU80_us_R | CTCTGCAATTGCATCCATCGC |
| KU80_ds_F | TTGAAATGAAAGTGAATTTATCGATATTGTAGG |
| KU80_ds_R | caaataggggttccgcggcggccgcATCAAACTCCCTATGTTCCTCGTCTTC |
| vector_F3 | AGACGAGGAACATAGGGAGTTTGATgcggccgccgcggaacccctatttg |
| vector_R3 | CCCATGAACACCTAGGTCGGAGAGCgcggccgctttccataggctccgcc |
| sNAT1_F | TTACCGTCTACCGCTGACGTTTTG |
| sNAT1_R | tcactttcatttcaaAGGAGTATAGAGTTGAATTTAATGGACG |
| 70486p_F3 | gatgcaattgcagagCTCGCTCTACTCTACTCTAGTCTAACTCTAGTTTAC |
| 70486t_R3 | caaattaaGGGAAGAGCAGGGTGGGC |
| TDH3p_F3 | tcttcccTTAATTTGCTGAAGCGGTTTGC |
| TDH3p_R3 | accaatcaatcagcataaggcccatTGCGAATGTGGATTAGAGTAAGATAGATAAC |
| TDH3t_F3 | tagtcggatcaagatcaaaaactaaGTGTGCGGTTGATGGTCTTCTATCTTC |
| TDH3t_R3 | agcggtagacggtaaATGTAGCGGGTGGTGATGGTG |
| MbBlh_F2 | tatcttactctaatccacattcgcaATGGGCCTTATGCTGATTGATTGGTG |
| MbBlh_R2 | agatagaagaccatcaaccgcacacTTAGTTTTTGATCTTGATCCGACTAGAATG |
| tgl4_us_F | ggcggagcctatggaaagcggccgcTTTCAACAAAAGATGCAGAAGAGCAG |
| tgl4_us_R | GGCCGCAATTACTGAGAGATATACCG |
| tgl4_ds_F | AGATATAAGTTAAACACCGTTTGAGTAATTAATATGGG |
| tgl4_ds_R | caaataggggttccgcggcggccgcCATTACCGTCAAATTCTTTATTAGATTCGG |
| vector_F4 | TCTAATAAAGAATTTGACGGTAATGgcggccgccgcggaacccctatttg |
| vector_R4 | TGCTCTTCTGCATCTTTTGTTGAAAgcggccgctttccataggctccgcc |
| TDH3p_F4 | tcagtaattgcggccTTAATTTGCTGAAGCGGTTTGC |
| TDH3p_R4 | TGCGAATGTGGATTAGAGTAAGATAGATAAC |
| TDH3t_F4 | GTGTGCGGTTGATGGTCTTCTATC |
| TDH3t_R4 | agcggtagacggtaaATGTAGCGGGTGGTGATGGTG |
| ble_F | TTACCGTCTACCGCTGACGTTTTG |
| ble_R | gtttaacttatatctAGGAGTATAGAGTTGAATTTAATGGACGTTG |
| HMG1_F | taatccacattcgcaATGACGAACTTCCTCGCGC |
| HMG1_R | ccatcaaccgcacacCTATGGCGAAATGCAAATCTGC |
| tHMG1_F | taatccacattcgcaATGGAGAAAATTATCGAAGTTCCTCAACC |
| tHMG1_R | ccatcaaccgcacacCTATGGCGAAATGCAAATCTGC |
| TDH3p_R5 | gaggaacttcgataattttctcCATTGCGAATGTGGATTAGAGTAAGATAGATAAC |
| KU80_us_R2 | gtagagtagagcgagCTCTGCAATTGCATCCATCGC |
| ACT1t_R | AGGAGTATAGAGTTGAATTTAATGGACGTTG |
| ble_F2 | gtataatatttcacaATGGCCAAGTTGACCAGTGC |
| ble_R2 | cggaacaacgtccgcTCAGTCCTGCTCCTCGGC |
| tHMG1_F2 | caactctatactcctTTAATTTGCTGAAGCGGTTTGC |
| tHMG1_R2 | tcactttcatttcaaATGTAGCGGGTGGTGATGGTG |
| natFw | tactctaatccacattcgcaATGGGTACCACTCTTGACGAC |
| natRv | gaagaccatcaaccgcacacacTTAGGGGCAGGGCATGCTC |
| vector(TDH3)Fw | GTGTGCGGTTGATGGTCTTC |
| vector(TDH3)Rv | TGCGAATGTGGATTAGAGTAAGATAGATAAC |
| Sh bleFw | tactctaatccacattcgcaATGGCCAAGTTGACCAGTG |
| Sh bleRv | gaagaccatcaaccgcacacacTCAGTCCTGCTCCTCGGC |
| hphFw | tactctaatccacattcgcaATGAAAAAGCCTGAACTCAC |
| hphRv | gaagaccatcaaccgcacacacGGTCCGCATCTACTCTATTC |
| KanRFw | tactctaatccacattcgcaATGAGCCATATTCAACGG |
| KanRRv | gaagaccatcaaccgcacacacTTAGAAAAACTCATCGAGC |
| LsLIG4_5'UTR_2k_Fw | gcccgcggccgcCTCAGACTTTACCACAGATACGG |
| LsLIG4_5'UTR_Rv | gagtagagcgagAGTTACCACAATTATATGCACATGGAGTC |
| 70486p(ERG10)Fw | aattgtggtaactCTCGCTCTACTCTACTCTAGTCTAAC |
| 70486p(ERG10)Rv | aagtgagaagcatTGTGAAAGTTGAATATAGATAGTAAGAGCTTTTTCG |
| LsLIG4_3'UTR_2k_Fw | ccctgctcttcccGCCTGTTATAGAAGTCAAGTTCGC |
| LsLIG4_3'UTR_2k_Rv | gggctgcaggaattcgatggggcccCACATGCACATCTTTATCGAGGAC |
| 70486t(ERG10)Fw | gagattttgtagACGTCGTCTCGCTTCCTCTC |
| 70486t(ERG10)Rv | agcaaattaatgGGGAAGAGCAGGGTGGGC |
| TDH3p(ERG13)Fw | ccctgctcttcccCATTAATTTGCTGAAGCGGTTTGC |
| TDH3p(ERG13)Rv | taggacgtgtcatTGCGAATGTGGATTAGAGTAAGATAGATAAC |
| TDH3p(ERG13)Fw | ataaaggagtaaGTGTGCGGTTGATGGTCTTC |
| TDH3p(ERG13)Rv | ggtagacggtaaTGATGGTGGAACAAAGTTGTTTTTAAGATC |
| 70486p(HMG1) Fw | actctatactcctCTCGCTCTACTCTACTCTAGTCTAAC |
| 70486p(HMG1) Rv | gaagttcgtcatTGTGAAAGTTGAATATAGATAGTAAGAGCTTTTTCG |
| 70486t(HMG1)Fw | atttcgccatagACGTCGTCTCGCTTCCTCTC |
| 70486t(HMG1) Rv | tctataacaggcGGGAAGAGCAGGGTGGGC |
| ERG10Fw | ttcaactttcacaATGGTTGCCAACCAGAACG |
| ERG10Rv | agcgagacgacgtCTACAAAATCTCAATTACAACAGCACTAGC |
| ERG13Fw | tccacattcgcaATGACACGTCCTACCGATATTGG |
| ERG13Rv | atcaaccgcacacTTACTCCTTTATAGCATACTCACGTCG |
| HMG1Fw | ttcaactttcacaATGACGAACTTCCTAGCGC |
| HMG1Rv | agcgagacgacgtCTATGGCGAAATGCAAATCTGC |
| P_sNAT1_T_Fw | tgttccaccatcaTTACCGTCTACCGCTGACG |
| P_sNAT1_T_Rv | gagtagagcgagAGGAGTATAGAGTTGAATTTAATGGACGTTG |
| VectorFw | GGGCCCCATCGAATTCCTGC |
| VectorRv | GGGCCCGGTACCCAGCTTTTG |
| ACT1_F | GCGGACGTTGTTCCGGATG |
| ACT1_R | TGTGAAATATTATACAATTAACTGTAGAAAGACAAAAATG |
| sNAT1_F2 | gtataatatttcacaATGGGTACCACTCTTGACGACAC |
| sNAT1_R2 | cggaacaacgtccgcTTAGGGGCAGGGCATGCTC |
| ScHMG1_us_F | ctatagggcgaattgattactcgagCGGCAAAAAAAGCGTTATCTTCTTTCTGTT |
| ScHMG1_us_R | GCTTGTTTTATGTATTTATCTACTTTGTATCA |
| ScHMG1_ds_F | atacataaaacaagcggatccACTTAGTCATACGTCATTGGTATTCTCTTGA |
| ScHMG1_ds_F | aaagggaacaaaagctgtaatctcgagGTTTCGGCCTCTCTTGCTCCATATGAGGGT |
| pBlue_F1 | CAGCTTTTGTTCCCTTTAGTGAGGGTTAATTG |
| pBlue_R1 | CAATTCGCCCTATAGTGAGTCGTATTACGCGC |
| PGK1p_F | ctatagggcgaattgtaccgttcgtatagcatacattatacgaagttatAGACGCGAATTTTTCGAAGAAGTACCTTCAAAGA |
| PGK1p_R | TGTTTTATATTTGTTGTAAAAAGTAGATAATTACT |
| PGK1t_F | ATTGAATTGAATTGAAATCGATAGATCAAT |
| PGK1t_R | aaagggaacaaaagctgggatcctaccgttcgtataatgtatgctatacgaagttatAAATTAAAACCGATTGACCAATATATGTCT |
| G418r_F1 | aacaaatataaaacaATGATTGAACAAGATGGATTGCACGCAGGTTCT |
| G418r_R1 | tcaattcaattcaatTCAGAAGAACTCGTCAAGAAGGCGATAGAA |
| pBlue_F2 | CAGCTTTTGTTCCCTTTAGTGAGGGTTAATTG |
| pBlue_R2 | CAATTCGCCCTATAGTGAGTCGTATTACGCGC |
| pBlue_HMG1_vec_F | tacgaacggtaggatccACTTAGTCATACGTCATTGGTATTCTCTTGAA |
| pBlue_HMG1_vec_R | GCTTGTTTTATGTATTTATCTACTTTGTATCAACA |
| G418r_F2 | taccgttcgtatagcatacattatacgaagttatAGACGCGAATTTTTCGAAGAAGTACCTTCA |
| G418r_R2 | ggatcctaccgttcgtataatgtatgctatacgaagttatAAATTAAAACCGATTGACCAATATATGTCTCT |
| ScHMG1_F | atacataaaacaagcAATAAAAAACACGCTTTTTCAGTTCGAGTTTATCA |
| ScHMG1_R | gctatacgaacggtaATCCTGGCGGAAAAAATTCATTTGTAAACT |
| pYESCT_F | TTGAAAAGCTAGCTTATCGATGATAAG |
| pYESCT_R | AAAACTGTATTATAAGTAAATGCATG |
| LEU2_F | ttataatacagttttTTAAGCAAGGATTTTCTTAACTTCTTC |
| LEU2_R | aagctagcttttcaaAACTGTGGGAATACTCAGGTATCG |
| HIS3_F | ttataatacagttttCAGCTTGTCTGTAAGCGGATGCCGGG |
| HIS3_R | aagctagcttttcaaAAAGCGCGCCTCGTTCAGAATGACACG |
| pYESCT_F2 | catcatcaccatcaccattgag |
| pYESCT_R2 | acagctgctagtagtccgatc |
| CsPDP_Sc opt_F | actactagcagctgtATGCATGATTGGCTGATTTTCCTAT |
| CsPDP_Sc opt_R | gtgatggtgatgatgTTTGGGACCTCTTTCGACATCTTG |
| LsGGS1_Sc opt_F | actactagcagctgtATGTCGACAGACGTCACGAGTAA |
| LsGGS1_Sc opt_R | gtgatggtgatgatgTGAAGAAGAATGCAAGCTGTCCAG |
| XdCrtE_Sc opt_F | actactagcagctgtATGGATTATGCGAACATATTGACAG |
| XdCrtE_Sc opt_R | gtgatggtgatgatgCAATGGAATATCTGCTAGTTTCTTC |
| lig4_us_check_F | GTAATCATAACGCATATGGCGCC |
| KanR_R | CGGAACAACGTCCGCTTAGAAAAACTCATCGAGCATCAAATG |
| KanR_F | GTATAATATTTCACAATGAGCCATATTCAACGGGAAAC |
| lig4_ds_check_R | CACCAATTTCACAATCTGATTCCGTC |
| tgl3_us_check_F | GGTATGACCGAGCCGACGATATTG |
| tgl3_ds_check_R | ATGACCGACGCAAGTGCAGC |
| 18S_check-us_F | ACTGCGAATGGCTCATTAAATCAGTTATC |
| Fu_MbBlh_R | GGTTCGAGAGGAAGCGAGACGACGTTTAGTTTTTGATCTTGATCCGACTAGAATG |
| Fu_MbBlh_F | TTACTATCTATATTCAACTTTCACAATGGGCCTTATGCTGATTGATTG |
| 18S_check-ds_R | TGCCCTTCCGTCAATTCCTTTAAG |
| ku80_check-us_F | TATTATTGGCGTTCTTGCGTTG |
| ble_R | CGGAACAACGTCCGCTCAGTCCTGCTCCTCGGC |
| ble_F | GTATAATATTTCACAATGGCCAAGTTGACCAGTGC |
| ku80_check-ds_R | GCATGCTTGCTCGAATACTGATTC |
| TGL4_check-us_F | AGTCGTTTGTTAGGCACCTGAGAAC |
| TGL4_check-ds_R | GCGCCAGTTTCTGTATAATGATCAG |

**Table S2 Gene sequences of codon-optimized *CsPDP*, *XdCrtE*, *McCarRP*, *McCarB,* *MbBlh, CrtZ* and *CrtW***

| Genes | Sequence |
| --- | --- |
| Codon-optimized *CsPDP* ORF for *S. cerevisiae* | ATGCATGATTGGCTGATTTTCCTATTACTCGTGGTTATAGAGGTCATTTTGTACGTGATACACCCGTTCAAACGGTATGCAGGCAAAGACATGATGACCGATTTGAGGTATCCACTACAGTCTAATACGGTTCCTGTTTGGGCAGTTCCCATGTATGCAATCTTATTGCCAATGCTGGTTTTTCTTGTCGTGTACTTTCGTAGGAGAGACATCTACGATTTGCATCATGCCATATTAGGGTTGTTTTACTCTGTACTTGTAACAGCCGTAATTACCGACTCGATTAAGAACGCTGTTGGTAGACCAAGACCTGACTTTTTCTGGAGATGTTTTCCAGATGGGAAAGATGTGTATGATCAACTAGGAAACGTGATATGTCACGGTGATAAAAACGTCATTAAGGAAGGTCACAAAAGCTTTCCCTCAGGACATACATCCTGGTCATTTGCTGGCTTAGGCTTTTTATCCCTGTATTTGTCTGGTAAGCTTAAGGCTTTCGATCGTAGAGGACATGTTGCTAAGTTATGCATCATCTTCCTACCGTTACTAGTTGCGTGTTTAGTCGGCATTTCAAGGGTAGATGACTATTGGCACCATTGGCAAGACGTATTTGCAGGTGGTTTGCTGGGTTTGACTGTCTCTACATTCTGCTATTTACAGTTCTTTCCTCCACCTTACCATCCACAAGGATGGGGTCCTTACGCTTATTTTCGCGTACTTGAGGAAAGTCATGGAAATACTCAAGCGAGTAGTGCCACTAACTTGCTCAATAGCGAATCTAGAGTTGGTGAAGAAGAGGAATCGAATGTTTTCATGGGGTTGCACTTAGCTCGAAATTCCTCTCTTCCAATGGAATCACATCAAGATGTCGAAAGAGGTCCCAAATAG |
| Codon-optimized *XdCrtE* ORF for *S. cerevisiae* | ATGGATTATGCGAACATATTGACAGCCATTCCCTTGGAATTTACCCCACAAGATGACATTGTCTTACTGGAACCATATCACTATCTAGGCAAAAATCCGGGTAAAGAGATTCGTTCACAACTAATAGAAGCCTTTAATTACTGGTTAGATGTGAAGAAAGAAGATCTCGAGGTGATCCAAAATGTGGTTGGAATGTTACATACTGCTAGCTTACTGATGGATGATGTAGAGGATTCATCTGTGTTGAGAAGAGGTTCACCAGTTGCCCATCTTATCTATGGTATTCCACAAACGATCAATACCGCAAATTACGTCTATTTCTTGGCTTATCAGGAAATCTTTAAGCTTAGACCAACACCAATTCCTATGCCTGTTATTCCTCCTAGTAGTGCTTCATTGCAATCATCTGTATCGTCCGCTTCTTCCTCATCATCGGCTTCTAGCGAAAATGGTGGTACTTCTACTCCCAATTCACAAATCCCATTCTCCAAAGATACCTACCTTGACAAGGTTATAACGGATGAGATGTTATCGCTACATAGGGGTCAAGGTCTGGAATTATTCTGGAGAGACAGTTTAACCTGTCCTTCTGAAGAAGAGTACGTTAAAATGGTTTTAGGGAAGACAGGAGGACTATTTAGGATAGCAGTAAGGCTGATGATGGCAAAGAGTGAGTGTGACATTGACTTTGTACAGCTTGTTAACTTAATATCCATCTACTTCCAAATTAGAGATGACTACATGAATCTACAGAGTAGCGAATATGCCCATAACAAGAATTTCGCAGAAGACTTGACAGAAGGCAAATTTTCCTTTCCCACTATCCACTCGATACACACGAACCCTAGTTCCCGCTTAGTCATTAACACTTTACAGAAGAAAAGCACATCTCCGGAAATTCTGCATCATTGCGTCAATTACATGCGAACAGAAACACACTCTTTCGAGTATACTAGAGAGGTTTTGAACACCTTATCTGGAGCACTTGAACGGGAATTGGGCAGATTGCAAGAAGAATTTGCTGAAGCGAATTCTCGTATGGATTTGGGTGATGTTGAGTCTGAAGGTAGAACTGGGAAAAACGTCAAACTCGAAGCTATATTGAAGAAACTAGCAGATATTCCATTGTAG |
| Codon-optimized *XdCrtE* ORF for *L. starkeyi* | ATGGACTATGCCAACATACTTACCGCTATTCCTCTCGAATTTACCCCTCAAGATGACATTGTGCTGTTGGAACCGTATCACTATCTTGGCAAAAATCCAGGGAAAGAGATAAGGAGCCAGTTGATAGAAGCCTTCAACTACTGGTTGGATGTGAAGAAGGAGGATTTGGAAGTGATTCAGAATGTAGTCGGAATGCTTCATACAGCATCTTTGCTTATGGATGACGTTGAAGACTCAAGTGTTCTTCGGAGAGGTTCACCGGTAGCACATCTTATCTACGGCATTCCTCAGACCATAAATACTGCCAATTACGTCTATTTCCTTGCATATCAGGAGATCTTCAAACTGAGGCCAACTCCGATCCCTATGCCCGTGATTCCACCTAGTTCTGCATCCCTCCAATCATCCGTCTCTTCAGCGTCTAGCTCTTCTTCTGCTTCCAGTGAAAATGGCGGTACATCCACTCCCAACTCGCAAATACCCTTTTCCAAAGACACATATCTGGACAAGGTTATTACGGATGAGATGCTATCGCTACATCGTGGTCAAGGTTTGGAGTTGTTTTGGAGAGATTCCCTCACATGTCCTTCCGAAGAAGAGTATGTGAAGATGGTGTTAGGGAAAACTGGAGGACTGTTTCGCATTGCGGTTCGACTCATGATGGCCAAATCAGAGTGCGATATCGACTTTGTTCAGCTAGTCAACTTGATCTCGATCTACTTCCAGATTCGAGACGATTACATGAACCTCCAAAGTTCCGAGTATGCTCACAACAAGAATTTTGCGGAGGACTTAACAGAGGGTAAGTTCTCATTTCCGACCATTCACTCTATCCATACGAATCCGAGCTCGAGATTGGTTATCAACACACTGCAAAAGAAGTCTACTAGTCCAGAGATTCTTCACCATTGCGTTAATTACATGCGGACAGAGACACATTCGTTCGAGTATACGCGAGAAGTTCTGAATACCCTTAGTGGAGCCTTGGAACGGGAATTGGGCCGATTGCAGGAAGAGTTCGCAGAGGCGAATTCGAGAATGGATCTAGGAGATGTCGAGAGCGAAGGTCGAACGGGCAAAAACGTCAAGTTAGAGGCTATCCTGAAAAAGCTCGCTGATATCCCGTTATAA |
| Codon-optimized *McCarRP* ORF for *L. starkeyi* | ATGTTGTTGACGTATATGGAAGTCCACTTGTATTATACCTTGCCGGTGCTTGGAGTTCTGAGCTGGTTGTCTCGACCCTACTACACAGCAACTGACGCCTTGAAGTTCAAGTTCTTGACATTGGTTGCGTTCACTACAGCAAGCGCATGGGATAACTATATCGTGTATCACAAAGCTTGGTCATATTGCCCTACTTGCGTGACGGCAGTCATAGGTTATGTACCACTCGAGGAGTACATGTTCTTCATTATCATGACCCTTCTAACTGTTGCTTTTACCAATCTGGTAATGCGGTGGCATTTGCACTCTTTCTTCATACGTCCTGAAACACCTGTCATGCAAAGCGTACTAGTCCGACTTGTGCCTATCACAGCCTTGTTGATTACTGCGTACAAAGCCTGGCATTTGGCCGTCCCTGGAAAACCGTTATTTTACGGCTCGTGCATCCTATGGTATGCGTGTCCGGTATTGGCCCTTTTATGGTTTGGAGCAGGTGAGTACATGATGAGAAGACCACTGGCCGTTCTCGTTTCGATAGCGTTGCCGACACTCTTCCTTTGTTGGGTGGATGTTGTGGCGATTGGCGCTGGTACTTGGGACATTTCGTTGGCAACATCGACAGGCAAATTTGTGGTGCCTCACCTTCCAGTGGAAGAGTTCATGTTCTTTGCACTGATTAACACAGTCTTGGTTTTTGGGACGTGTGCTATCGACCGAACGATGGCCATTCTGCATCTGTTCAAGAACAAATCACCATATCAGCGCCCCTATCAGCACTCAAAATCCTTTCTTCACCAGATCCTCGAGATGACATGGGCTTTCTGCCTACCGGACCAAGTCCTTCATTCCGATACCTTCCATGATCTTTCAGTGTCCTGGGACATTCTCCGCAAAGCGTCTAAGTCCTTTTACACAGCGAGTGCTGTTTTTCCGGGTGATGTTCGGCAGGAGTTGGGCGTACTTTACGCTTTTTGCAGAGCTACTGATGACCTTTGCGATAATGAGCAAGTTCCCGTCCAAACCCGTAAGGAACAGCTGATCCTAACTCACCAGTTCGTCAGCGACCTCTTTGGGCAGAAAACGTCCGCTCCAACAGCCATTGACTGGGACTTTTACAACGATCAACTTCCCGCATCTTGCATCTCTGCTTTTAAGTCGTTTACCAGACTCCGACATGTCCTGGAAGCTGGCGCAATTAAGGAGCTACTAGATGGGTATAAATGGGATTTGGAACGAAGGTCGATTCGAGATCAGGAAGATTTGCGGTACTATTCCGCCTGTGTTGCCAGTTCTGTGGGAGAAATGTGCACAAGGATCATACTGGCGCATGCAGACAAGCCAGCATCTCGACAGCAGACACAATGGATCATCCAGAGAGCCAGAGAGATGGGCTTAGTTTTACAGTACACCAATATAGCTCGGGATATCGTCACTGATAGTGAGGAACTGGGTCGATGCTATCTTCCCCAAGACTGGTTAACGGAGAAGGAGGTTGCACTTATCCAAGGTGGTTTGGCAAGGGAGATTGGAGAGGAACGTCTTCTCTCACTCTCACATAGACTGATATACCAAGCTGATGAACTGATGGTTGTTGCCAATAAGGGAATTGACAAGTTGCCTAGTCATTGCCAAGGCGGTGTTAGAGCGGCTTGCAATGTCTACGCGTCTATTGGAACCAAGCTAAAGTCCTATAAGCACCATTATCCGAGTCGAGCGCATGTGGGAAACTCTAAAAGGGTGGAGATTGCCTTACTTTCCGTCTATAATCTCTATACAGCACCTATTGCAACCAGTAGTACCACACATTGTCGCCAGGGCAAAATGCGGAATCTGAATACGATATAA |
| Codon-optimized *McCarB* ORF for *L. starkeyi* | ATGTCCAAAAAGCACATAGTCATCATTGGTGCAGGTGTTGGTGGAACAGCCACTGCTGCACGCTTAGCTCGAGAAGGCTTCAAGGTGACGGTAGTGGAGAAGAACGATTTTGGAGGTGGAAGATGCTCTTTAATCCATCATCAGGGCCATCGTTTCGACCAAGGACCTTCGCTGTACCTCATGCCGAAGTACTTTGAGGATGCATTTGCGGATTTGGACGAAAGGATTCAGGATCACCTTGAGTTACTGAGGTGCGACAACAACTACAAAGTGCATTTTGACGATGGTGAGTCTATACAGCTGTCTTCTGATCTGACTCGAATGAAAGCGGAATTGGATAGGGTTGAGGGGCCCTTGGGATTTGGCCGGTTTCTGGATTTCATGAAGGAAACACACATACACTACGAAAGTGGCACACTCATCGCCCTTAAGAAGAACTTTGAGTCAATTTGGGACCTTATCCGGATCAAATATGCACCTGAGATCTTTCGACTTCACCTCTTCGGCAAGATCTATGACAGAGCCAGTAAGTACTTCAAGACCAAGAAAATGCGTATGGCGTTTACCTTTCAGACCATGTATATGGGGATGAGTCCGTATGATGCTCCTGCAGTGTATTCACTTCTGCAGTACACAGAGTTTGCCGAAGGAATTTGGTATCCAAGAGGAGGGTTTAATATGGTTGTGCAAAAGTTGGAGGCGATTGCCAAGCAGAAATATGACGCGGAGTTTATATACAATGCTCCTGTGGCCAAAATCAACACGGACGATGCTACCAAACAAGTTACGGGTGTCACGTTGGAGAATGGTCACATTATCGACGCAGATGCAGTCGTATGCAATGCAGACCTCGTTTACGCATATCACAATCTCCTTCCTCCCTGTAGATGGACCCAGAACACCTTGGCATCAAAGAAACTAACTAGCTCCTCGATCTCCTTCTATTGGTCGATGAGCACTAAGGTTCCCCAACTAGATGTCCATAATATCTTCTTGGCGGAAGCATATCAGGAATCGTTTGACGAGATTTTCAAAGATTTCGGTCTTCCATCTGAAGCCAGTTTCTATGTCAATGTCCCGAGCAGAATTGATCCGTCTGCTGCTCCTGATGGTAAGGATAGTGTCATTGTGCTCGTTCCAATAGGCCATATGAAGTCCAAAACAGGCGATGCCTCTACTGAGAATTATCCGGCGATGGTGGATAAAGCTCGCAAGATGGTTCTAGCGGTAATTGAGCGACGATTAGGCATGTCCAACTTCGCAGACTTGATTGAGCATGAGCAGGTTAACGACCCAGCCGTTTGGCAGTCCAAGTTCAATCTTTGGAGAGGCTCCATTCTTGGCCTAAGTCATGATGTGTTGCAAGTGCTGTGGTTTAGACCGTCAACGAAGGACTCAACAGGACGATATGACAATCTGTTTTTCGTTGGTGCTTCTACCCATCCGGGAACTGGCGTACCCATTGTGTTGGCCGGGAGCAAATTGACATCAGACCAGGTTGTCAAATCTTTTGGTAAGACACCGAAACCTCGGAAAATCGAAATGGAGAATACACAAGCTCCACTAGAAGAACCTGATGCAGAGTCCACATTTCCTGTCTGGTTTTGGCTTCGAGCTGCGTTCTGGGTTATGTTCATGTTTTTCTACTTCTTCCCCCAAAGTAATGGACAAACACCAGCTTCGTTCATAAACAACTTGTTGCCAGAAGTTTTCCGGGTCCATAATTCGAATGTCATTTAA |
| Codon-optimized *MbBlh* ORF for *L. starkeyi* | ATGGGCCTTATGCTGATTGATTGGTGTGCATTGGCACTTGTGGTCTTTATTGGACTTCCACATGGGGCTTTGGATGCAGCTATCTCTTTTTCGATGATATCTTCAGCCAAACGAATCGCTAGATTGGCGGGTATTCTGTTGATTTACCTTCTTCTCGCAACTGCCTTCTTCTTGATATGGTATCAACTCCCTGCCTTTTCGCTCCTGATTTTCTTACTGATCTCGATCATCCACTTTGGCATGGCAGACTTCAATGCTAGTCCGTCAAAACTCAAATGGCCTCATATCATTGCCCATGGTGGTGTTGTGACTGTGTGGTTACCGCTTATTCAGAAGAATGAGGTAACAAAGCTCTTCTCCATTCTAACAAATGGACCTACACCCATTCTGTGGGATATCCTGTTGATATTCTTCCTTTGCTGGTCTATTGGCGTTTGCCTACATACGTACGAAACCCTCCGTAGTAAGCACTATAACATCGCGTTTGAGTTGATAGGCTTGATTTTTCTTGCTTGGTATGCTCCACCCTTAGTCACGTTTGCCACCTATTTTTGCTTCATACATTCCAGAAGGCACTTTAGCTTCGTTTGGAAACAGTTGCAACACATGAGTTCCAAGAAGATGATGATTGGTTCAGCCATCATACTGTCTTGTACCTCCTGGCTAATTGGAGGTGGGATCTACTTTTTCCTAAACAGCAAGATGATTGCGTCAGAAGCAGCGTTACAGACTGTCTTCATTGGATTGGCAGCGTTGACAGTTCCGCATATGATCCTTATAGACTTCATCTTTCGACCACATTCTAGTCGGATCAAGATCAAAAACTAA |
| Codon-optimized *CrCrtW (CrtW* derived from *Chlamydomonas reinhardtii)* ORF for *L. starkeyi* | ATGGGACCAGGCATTCAGCCGACAAGTGCTAGACCATGTAGCCGTACAAAGCACAGTAGATTCGCACTACTGGCCGCCGCCCTTACAGCCCGTCGAGTCAAGCAGTTCACGAAGCAGTTTCGATCTCGAAGGATGGCTGAGGACATCCTCAAGCTTTGGCAACGGCAATACCATCTTCCGAGAGAGGATTCCGATAAGCGGACTCTCCGCGAAAGAGTCCATCTCTATCGACCTCCCAGATCTGATCTTGGGGGCATAGCCGTCGCCGTTACGGTTATTGCGCTGTGGGCTACCTTGTTTGTGTATGGCTTATGGTTCGTGAAGCTACCTTGGGCGCTCAAAGTCGGCGAGACAGCGACATCTTGGGCGACAATTGCAGCGGTGTTCTTTTCCCTGGAGTTCTTGTATACCGGCTTGTTCATCACAACGCATGATGCCATGCACGGGACCATTGCATTACGGAATCGACGCCTTAACGACTTTCTTGGCCAACTTGCTATCAGCCTGTATGCATGGTTCGACTATTCGGTTCTTCACCGAAAACATTGGGAACACCACAACCATACGGGAGAACCTCGGGTAGATCCGGATTTCCATCGAGGAAATCCCAATCTGGCCGTGTGGTTTGCCCAGTTTATGGTGTCTTACATGACACTCTCACAGTTCTTGAAGATTGCGGTCTGGTCAAACCTGTTGCTGTTGGCCGGTGCTCCGTTAGCAAATCAGCTTCTGTTTATGACCGCTGCACCTATCCTATCGGCGTTCAGGTTGTTTTACTACGGTACTTATGTTCCCCATCACCCAGAGAAAGGGCATACCGGAGCTATGCCTTGGCAGGTTTCCAGGACATCTTCGGCGAGTAGACTTCAGAGTTTCCTAACCTGCTACCACTTTGACTTGCATTGGGAGCATCATCGATGGCCGTATGCACCGTGGTGGGAACTACCCAAATGCCGACAGATAGCAAGAGGAGCCGCGTTGGCACCAGGTCCTTTGCCCGTACCAGCCGCAGCAGCAGCAACTGCGGCCACTGCGGCAGCAGCCGCTGCTGCTACTGGCTCACCAGCACCAGCAAGCAGGGCTGGTTCAGCCTCTTCCGCGTCAGCTGCTGCAAGTGGCTTTGGTAGCGGACACTCTGGTTCGGTTGCAGCTCAACCGTTGTCGTCCTTGCCTCTCCTGAGTGAAGGTGTCAAAGGGTTAGTGGAGGGAGCCATGGAGCTTGTTGCTGGGGGTTCCTCCTCTGGAGGTGGTGGCGAAGGTGGAAAACCTGGCGCTGGAGAGCATGGATTGTTGCAAAGACAACGGCAACTCGCTCCGGTCGGCGTTATGGCGTAG |
| Codon-optimized *EuCrtZ (CrtZ* derived from *Erwinia uredovora)* ORF for *L. starkeyi* | ATGTTGTGGATTTGGAACGCATTGATCGTCTTCGTTACGGTCATTGGGATGGAGGTGATAGCCGCTTTAGCGCACAAGTACATCATGCATGGGTGGGGATGGGGATGGCATCTTTCACACCACGAACCACGCAAAGGTGCGTTTGAGGTCAATGACCTGTATGCGGTAGTGTTTGCTGCCCTCTCCATTCTCCTGATCTATCTTGGCAGTACTGGCATGTGGCCGTTACAGTGGATTGGTGCTGGTATGACCGCCTATGGCCTACTGTACTTCATGGTGCATGATGGCCTTGTTCACCAGAGATGGCCATTTCGGTACATACCCCGAAAAGGCTACCTAAAGCGACTCTATATGGCCCATCGGATGCATCATGCAGTTCGTGGAAAAGAGGGTTGCGTTTCGTTCGGCTTCTTGTATGCACCGCCTTTGAGCAAGTTGCAAGCGACACTTCGAGAGAGGCATGGAGCTAGAGCAGGAGCAGCTAGAGATGCACAAGGTGGTGAAGACGAACCTGCCTCTGGAAAGTAG |
| Codon-optimized *HpCrtZ (CrtZ* derived from *Haematococcus pluvialis)* ORF for *L. starkeyi* | ATGTTGTCCAAGTTGCAGTCTATCTCCGTGAAAGCTAGACGAGTCGAGTTGGCTAGGGACATTACTCGACCGAAAGTATGCCTCCATGCCCAGAGATGTAGTCTAGTCCGTCTCCGTGTAGCAGCTCCGCAAACGGAGGAAGCAGTTGGAACGCAGCAAGCGGCAGGAGCCGGTGATGAGCATTCAGCAGACGTTGCACTTCAGCAGCTTGATCGGGCCATTGCAGAACGGAGAGCCAGACGAAAGCGAGAGCAACTGTCGTATCAGGCTGCTGCGATAGCTGCGTCTATCGGCGTTTCAGGCATTGCCATCTTTGCGACATACCTGCGCTTTGCGATGCACATGACAGTCGGTGGTGCAGTTCCTTGGGGAGAGGTTGCGGGCACTCTACTTCTCGTGGTTGGTGGTGCGTTAGGAATGGAGATGTATGCACGATATGCCCACAAAGCCATTTGGCATGAGTCTCCATTGGGCTGGCTACTGCACAAGTCGCATCATACACCGAGAACAGGTCCCTTTGAAGCCAACGACTTGTTCGCCATCATCAATGGGTTACCAGCCATGCTGCTTTGCACCTTTGGCTTCTGGTTGCCTAATGTCCTGGGTACTGCTTGCTTTGGAGCTGGATTGGGCATAACCCTGTATGGTATGGCGTACATGTTCGTGCATGATGGCCTTGTGCATCGAAGATTCCCCACAGGGCCAATTGCTGGCTTGCCCTACATGAAGCGCCTTACCGTTGCACATCAGCTTCATCACAGTGGGAAGTATGGGGGTGCACCTTGGGGAATGTTCCTCGGACCGCAAGAGCTCCAACACATACCTGGCGCAGCTGAAGAGGTGGAACGGTTAGTCTTGGAACTTGATTGGAGCAAAAGGTAG |
| Codon-optimized *BrevCrtZ (CrtZ* derived from *Brevundimonas* sp. SD212*)* ORF for *L. starkeyi* | ATGTCTGCGGTAACGCCAATGAGTCGTGTTGTGCCGAATCAAGCCTTGATTGGACTAACGCTTGCAGGTCTGATAGCTGCTGCTTGGTTAACGCTTCACATCTATGGCGTCTACTTCCATCGGTGGACTATCTGGTCTGTGCTCACAGTTCCCCTCATTGTTGCAGGTCAGACATGGTTGTCAGTGGGGTTGTTCATAGTCGCGCATGATGCGATGCATGGCTCGCTAGCACCTGCAAGACCTCGATTGAACACTGCCATTGGGTCCTTAGCTCTTGCGTTATATGCCGGCTTTCGATTTACCCCACTAAAGACAGCGCACCATGCACATCATGCTGCTCCTGGAACAGCTGATGATCCGGACTTTCACGCTGATGCTCCTAGGGCCTTTTTGCCGTGGTTCTATGGCTTCTTTCGGACCTACTTCGGTTGGAGAGAACTCGCAGTCTTGACAGTGCTTGTTGCCGTTGCCGTACTCATCCTTGGAGCCAGAATGCCGAATCTGCTCGTCTTTTGGGCGGCACCTGCACTGCTTTCGGCATTGCAGCTTTTCACCTTCGGTACTTGGTTGCCACATCGCCATACCGACGATGCATTTCCGGACAACCACAATGCCAGGACATCCCCCTTTGGTCCCGTCCTGTCACTTCTGACTTGCTTTCACTTCGGACGACATCACGAGCATCACCTGACACCTTGGAAACCATGGTGGAGCTTGTTCAGTTAG |
| Codon-optimized *PspCrtW (CrtW* derived from *Paracoccus* sp. N81106*)* ORF for *L. starkeyi* | ATGTCAGCGCATGCTCTTCCGAAAGCTGACCTAACAGCGACATCGTTGATCGTTTCTGGAGGCATTATTGCCGCCTGGTTGGCACTGCACGTTCATGCTCTCTGGTTTTTGGATGCTGCAGCTCACCCTATCCTCGCAATTGCCAACTTCCTTGGTTTGACCTGGCTTAGTGTAGGCCTGTTCATCATCGCCCATGATGCGATGCACGGAAGTGTGGTTCCTGGTAGACCTCGAGCAAATGCAGCAATGGGCCAATTGGTCCTATGGCTCTATGCTGGTTTCTCTTGGCGAAAGATGATCGTCAAGCATATGGCACATCACAGACATGCCGGTACAGACGATGATCCGGACTTTGACCATGGTGGACCAGTTCGATGGTATGCGAGGTTCATAGGGACCTACTTTGGATGGAGGGAAGGTCTGCTTTTGCCCGTCATTGTGACGGTGTACGCGTTGATACTAGGGGATCGGTGGATGTACGTCGTGTTTTGGCCATTACCCTCCATTCTCGCTTCGATTCAGTTGTTCGTCTTTGGAACCTGGTTACCCCATCGTCCAGGACACGATGCATTCCCTGATCGGCATAATGCCCGATCATCCCGCATAAGCGACCCGGTTTCTCTGCTGACTTGCTTCCATTTTGGCGGCTATCACCACGAGCATCACCTTCATCCGACAGTACCTTGGTGGAGACTTCCATCCACGAGAACTAAAGGCGACACTGCCTAG |
| Codon-optimized *McCarRP(n)* ORF for *L. starkeyi* | ATGCTTCTCACTTACATGGAGGTTCACCTTTACTACACTCTTCCTGTCTTGGGTGTCTTGTCTTGGCTTTCTCGTCCTTATTATACCGCTACTGACGCTCTTAAGTTCAAGTTCCTCACCCTTGTCGCTTTCACTACCGCTTCCGCTTGGGACAACTACATCGTCTACCACAAGGCTTGGTCCTACTGCCCTACTTGCGTCACCGCTGTCATTGGTTACGTCCCTCTTGAGGAGTACATGTTCTTCATCATTATGACTCTCCTTACCGTCGCTTTCACCAACCTTGTCATGCGTTGGCACCTTCACTCCTTCTTCATTCGTCCTGAGACTCCTGTCATGCAGTCCGTTCTTGTCCGTCTTGTTCCTATCACTGCTCTTCTCATCACTGCTTACAAGGCTTGGCACCTTGCTGTTCCTGGTAAACCTCTCTTCTACGGTTCCTGCATCCTTTGGTACGCTTGCCCTGTTCTCGCTCTTCTCTGGTTCGGTGCTGGTGAGTACATGATGCGTCGTCCTCTTGCTGTTCTTGTCTCCATTGCTCTCCCTACTCTTTTCCTCTGCTGGGTCGATGTTGTCGCTATCGGTGCTGGTACTTGGGACATCTCCCTTGCTACTTCCACTGGTAAATTCGTTGTCCCTCACCTTCCTGTTGAGGAGTTCATGTTCTTCGCTCTTATCAACACTGTCCTCGTTTTCGGTACCTGCGCTATCGATCGTACTATGGCTATTCTCCACCTTTTCAAGAACAAGTCCCCTTACCAGCGTCCTTACCAGCACTCCAAGTCCTTCCTTCACCAGATCCTTGAGATGACTTGGGCTTTCTGCCTTCCTGATCAGGTCCTCCACTCCGACACCTTCCACGATCTCTCCGTCTCCTGGGACATTCTCCGTAAGGCTTCCAAGTCCTTCTACACTGCTTCCGCTGTTTTCCCTGGTGACGTGCGTCAGGAGCTTGGTGTCCTTTACGCTTTCTGCCGTGCTACTGACGATCTCTGCGACAACGAGCAGGTCCCTGTTCAGACTCGTAAGGAGCAGCTTATCCTCACTCACCAGTTCGTCTCCGACCTTTTCGGTCAGAAGACTTCCGCTCCTACTGCTATCGATTGGGACTTCTACAACGACCAGCTTCCTGCTTCCTGCATTTCCGCTTTCAAGTCCTTCACTCGTCTCCGTCACGTTCTTGAGGCTGGTGCTATCAAGGAGCTCCTTGATGGTTACAAGTGGGACCTTGAGCGTCGTTCCATCCGTGACCAGGAGGATCTTCGTTACTACTCCGCTTGCGTTGCTTCCTCCGTCGGTGAGATGTGCACTCGTATCATTCTCGCTCACGCTGATAAGCCTGCTTCCCGTCAGCAGACTCAGTGGATTATCCAGCGTGCTCGTGAGATGGGTCTCGTTCTTCAGTACACCAACATTGCTCGTGACATCGTCACCGATTCCGAGGAGCTCGGTCGTTGCTACCTCCCTCAGGATTGGCTCACTGAGAAGGAGGTCGCTCTCATTCAGGGTGGTCTTGCTCGTGAGATTGGTGAGGAGCGTCTTCTCTCCCTTTCCCACCGTCTCATCTACCAGGCTGATGAGCTTATGGTCGTTGCTAACAAGGGTATCGATAAGCTTCCTTCCCACTGCCAGGGTGGTGTTCGTGCTGCTTGCAACGTCTACGCTTCCATTGGTACCAAGCTCAAGTCCTACAAGCACCACTACCCTTCCCGTGCTCACGTTGGTAACTCCAAGCGTGTTGAGATCGCTCTTCTCTCCGTCTACAACCTCTACACCGCTCCTATCGCTACTTCGTCTACTACTCACTGCCGTCAGGGTAAGATGCGTAACCTCAACACTATCTAA |
| Codon-optimized *McCarB(n)* ORF for *L. starkeyi* | ATGTCTAAGAAGCACATCGTTATTATCGGTGCCGGTGTCGGTGGTACTGCCACTGCCGCCCGTCTCGCCCGTGAGGGTTTCAAGGTCACTGTTGTCGAGAAGAACGACTTCGGTGGTGGTCGTTGCTCCCTCATTCACCACCAGGGTCACCGTTTCGACCAGGGTCCTTCCCTTTACCTCATGCCTAAGTACTTCGAGGACGCTTTCGCTGACCTTGATGAGCGTATTCAGGACCACCTTGAGCTTCTCCGTTGCGACAACAACTACAAGGTCCACTTCGATGACGGTGAGTCCATCCAGCTCTCCTCCGATCTCACCCGTATGAAGGCTGAGCTTGATCGTGTCGAGGGTCCTCTTGGTTTCGGTCGTTTCCTCGATTTCATGAAGGAGACTCACATCCACTACGAGTCCGGTACTCTTATCGCTCTCAAGAAGAACTTCGAGTCCATCTGGGACCTCATTCGTATCAAGTACGCTCCTGAGATCTTCCGTCTCCACCTTTTCGGTAAAATCTACGATCGTGCTTCCAAGTACTTCAAGACTAAGAAGATGCGTATGGCTTTCACCTTCCAGACTATGTACATGGGTATGTCCCCTTACGATGCTCCTGCTGTTTACTCCCTTCTCCAGTACACTGAGTTCGCTGAGGGTATCTGGTACCCTCGTGGTGGTTTCAACATGGTTGTCCAGAAGCTTGAGGCTATCGCTAAGCAGAAGTACGACGCTGAGTTCATCTACAACGCTCCTGTCGCTAAGATCAACACTGACGATGCTACTAAGCAGGTTACTGGTGTCACTCTCGAGAACGGTCACATCATTGACGCTGATGCTGTCGTTTGCAACGCTGATCTTGTCTACGCTTACCACAACCTCCTTCCTCCTTGCCGTTGGACCCAGAACACTCTCGCTTCCAAGAAGCTTACTTCCTCCTCCATTTCCTTCTACTGGTCCATGTCCACTAAGGTCCCTCAGCTTGATGTCCACAACATCTTCCTCGCTGAGGCTTACCAGGAGTCCTTCGACGAGATCTTCAAGGACTTCGGTCTTCCTTCCGAGGCTTCCTTCTACGTCAACGTCCCTTCCCGTATTGACCCTTCCGCTGCTCCTGACGGTAAAGATTCCGTCATCGTTCTCGTCCCTATTGGTCACATGAAGTCCAAGACTGGTGACGCTTCCACTGAGAACTACCCTGCTATGGTCGATAAGGCTCGTAAGATGGTCCTTGCTGTTATCGAGCGTCGTCTTGGTATGTCCAACTTCGCTGATCTCATTGAGCACGAGCAGGTTAACGACCCTGCTGTTTGGCAGTCCAAGTTCAACCTTTGGCGTGGTTCCATTCTCGGTCTTTCCCACGATGTTCTTCAGGTCCTCTGGTTCCGTCCTTCCACCAAGGATTCCACCGGTCGTTACGACAACCTCTTCTTCGTTGGTGCTTCCACTCACCCTGGTACCGGTGTCCCTATCGTTCTTGCTGGTTCCAAGCTCACTTCCGACCAGGTCGTTAAGTCCTTCGGTAAAACCCCTAAGCCTCGTAAGATCGAGATGGAGAACACTCAGGCTCCTCTTGAGGAGCCTGATGCTGAGTCCACTTTCCCTGTTTGGTTCTGGCTCCGTGCTGCTTTCTGGGTCATGTTCATGTTCTTCTACTTCTTCCCTCAGTCCAACGGTCAGACCCCTGCTTCTTTCATCAACAACTTGCTTCCTGAGGTTTTCCGTGTCCACAACTCTAACGTCATCTAA |
| Codon-optimized *ScENV9* ORF for *L. starkeyi* | ATGTTAGATCCCAGAATCCTTCCCTACTATGATCCAGCTGTCGAACGGAAAATTGCCGTCGTTACCGGAGGGAATACAGGCATTGGATGGTATACTGTCCTGCATCTTTACCTACATGGCTTTGTAGTGTATATTTGCGGAAGGAATAGTCACAAAATCTCCAAAGCCATTCAGGAGATTCTCGCTGAAGCGAAGAAACGGTGCCATGAGGATGATGACGGATCATCTCCAGGTGCAGGACCTGGTCCATCTATTCAGAGACTTGGCTCTCTTCACTATATTCACCTTGATCTGACAGACCTCAAATGCGTTGAGAGAGCTGCGTTGAAGATCCTGAAACTAGAGGACCATATCGACGTTCTGGTGAACAATGCTGGCATTATGGCTGTTCCTCTCGAAATGACCAAGGATGGTTTTGAGGTTCAGTTGCAAACGAACTATATCTCGCACTTTATCTTCACGATGCGATTGCTTCCTCTATTGCGTCATTGTCGAGGTCGAATCATTTCACTCTCTTCCATAGGTCATCATCTCGAGTTTATGTACTGGAAGCTATCCAAGACTTGGGACTATAAGCCGAATATGCTGTTTACCTGGTTTCGATATGCAATGAGTAAAACCGCCTTGATACAGTGCACGAAGATGTTAGCCATTAAGTATCCGGATGTGTTGTGCCTGTCTGTTCACCCTGGGTTAGTCATGAACACTAACCTGTTCTCCTACTGGACAAGGCTTCCGATAGTAGGAATCTTCTTTTGGTTGTTGTTCCAAGTGGTTGGGTTCTTCTTCGGAGTCTCCAATGAGCAAGGTAGTTTGGCAAGTCTTAAGTGTGCGTTGGACCCGAATTTGAGCGTGGAAAAAGACAATGGCAAGTACTTCACTACAGGCGGTAAAGAGTCAAAGAGCTCATACGTCTCGAATAACGTGGATGAAGCAGCATCGACATGGATCTGGACAGTTCATCAGCTTCGCGATAGAGGCTTTGATATATAA |

**Table S3 Genes and primers for quantitative real-time PCR in this study**

| Gene | Forward primer sequence (5'-3') | Reverse primer sequence (5'-3') |
| --- | --- | --- |
| *ACT1* | TGAAGCTGCTGGCATTCATG | TCTGCATTCTGTCGGCAATG |
| *MbBlh* | TCTTCAGCCAAACGAATCGC | TCAAGAAGAAGGCAGTTGCG |

**Table S4 *Lipomyces starkeyi* homologues in mevalonate, β-carotene synthesis, and vitamin A synthesis pathways**

| *Lipomyces starkeyi*  (Transcript ID) | *Saccharomyces cerevisiae* | Identity (%) | Similarity (%) |
| --- | --- | --- | --- |
| Erg10p (285732) | Erg10p | 65.9 | 78.9 |
| Erg13p (63385) | Erg13p | 47.2 | 63.2 |
| Hmg1p (5675) | Hmg1p | 45.6 | 60.2 |
| Erg12p (54023) | Erg12p | 39.4 | 59.8 |
| Erg8p (37388) | Erg8p | 38.3 | 52.3 |
| Mvd1p (1591) | Mvd1p | 60.4 | 71.7 |
| Idi1p (5292) | Idi1p | 50.0 | 62.8 |
| Erg20p (69224) | Erg20p | 64.2 | 76.7 |
| Ggs1p (1857) | Bts1p | 36.0 | 55.0 |
| Env9p (7299) | Env9p | 35.7 | 51.5 |

Homologues of *L. starkeyi* mevalonate pathway-related enzymes (Erg10p, Erg13p, Hmg1p, Erg12p, Erg8p, Mvd1p, Idi1p and Erg20p), β-carotene synthesis pathway-related enzyme (Ggs1p), and vitamin A synthesis pathway-related enzyme (Env9p) were searched in the BLASTP analysis using the amino acid sequences of Erg10p, Erg13p, Hmg1p, Erg12p, Erg8p, Mvd1p, Idi1p, Erg20p, Ggs1p, and Env9p in *S. cerevisiae*. Pairwise sequence alignments between *L. starkeyi* and *S. cerevisiae* were performed using EMBOSS Needle program [1].

**Table S5 Phenotypic comparison of *LsTGL3* and *LsTGL4* double knockout strains with the control strain ∆*lslig4***

| Strain | Cell concentration  (× 10^8^ cells/mL) | | Glucose concentration (g/L) | | Amount of TAG per cell  (mg/10^8^ cells) | |
| --- | --- | --- | --- | --- | --- | --- |
|  | Day 1 | Day 3 | Day 1 | Day 3 | Day 1 | Day 3 |
| ∆*lslig4* | 0.89 _±0.02_ | 1.82 _±0.05_ | 50.1 _±1.61_ | 23.9 _±1.91_ | 0.40 _±0.02_ | 1.28 _±0.07_ |
| ∆*lstgl3*∆*lstgl4* | 0.85 _±0.05_ | 1.72 _±0.12_ | 50.9 _±1.11_ | 22.8 _±2.36_ | 0.39 _±0.06_ | 1.13 _±0.08_ |

Culture conditions: The strains were cultured in 500 mL baffled flasks with 300 mL of S medium (5.8% glucose) at 30°C.

**Supplementary Figures**

**
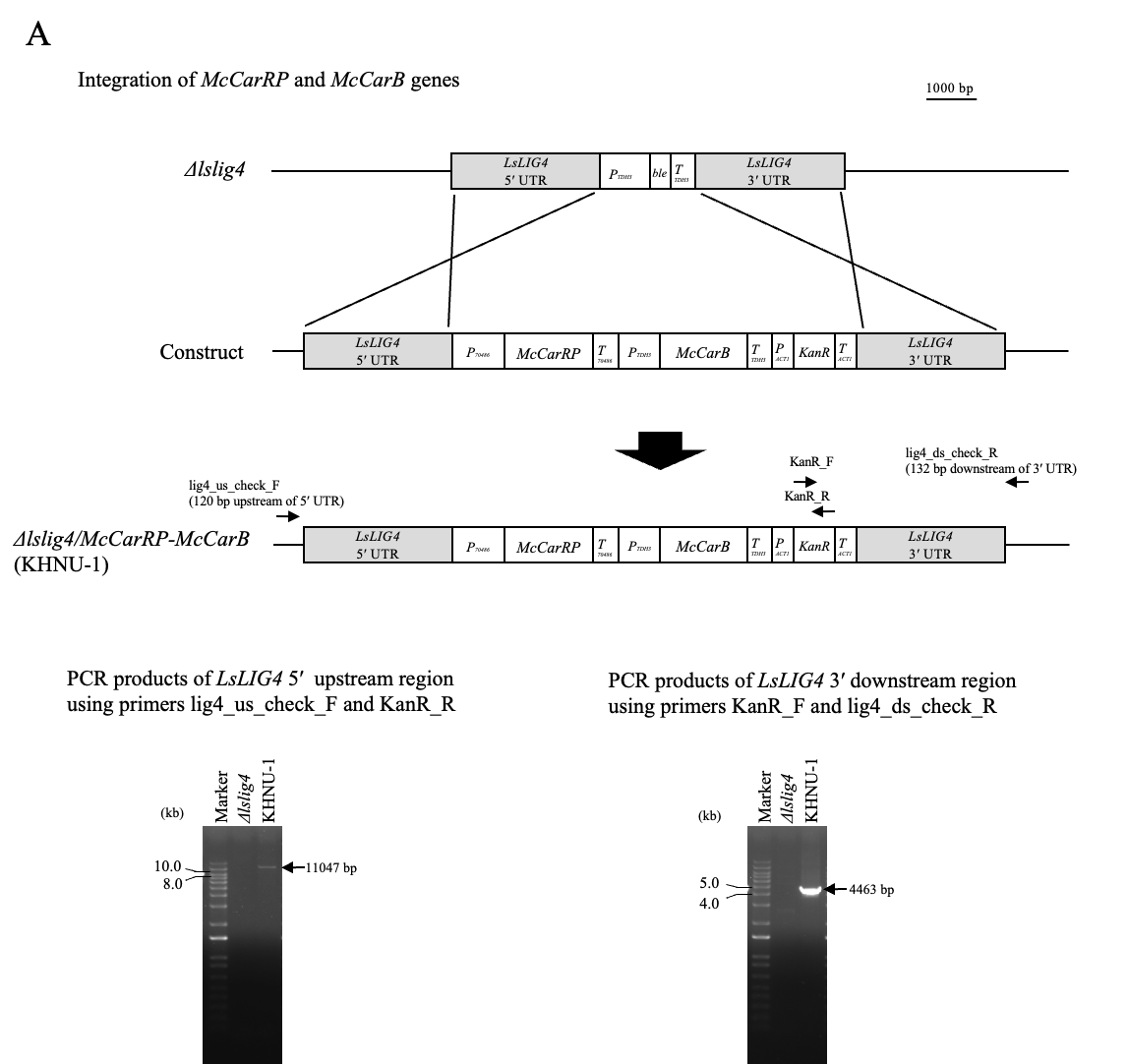
**

**
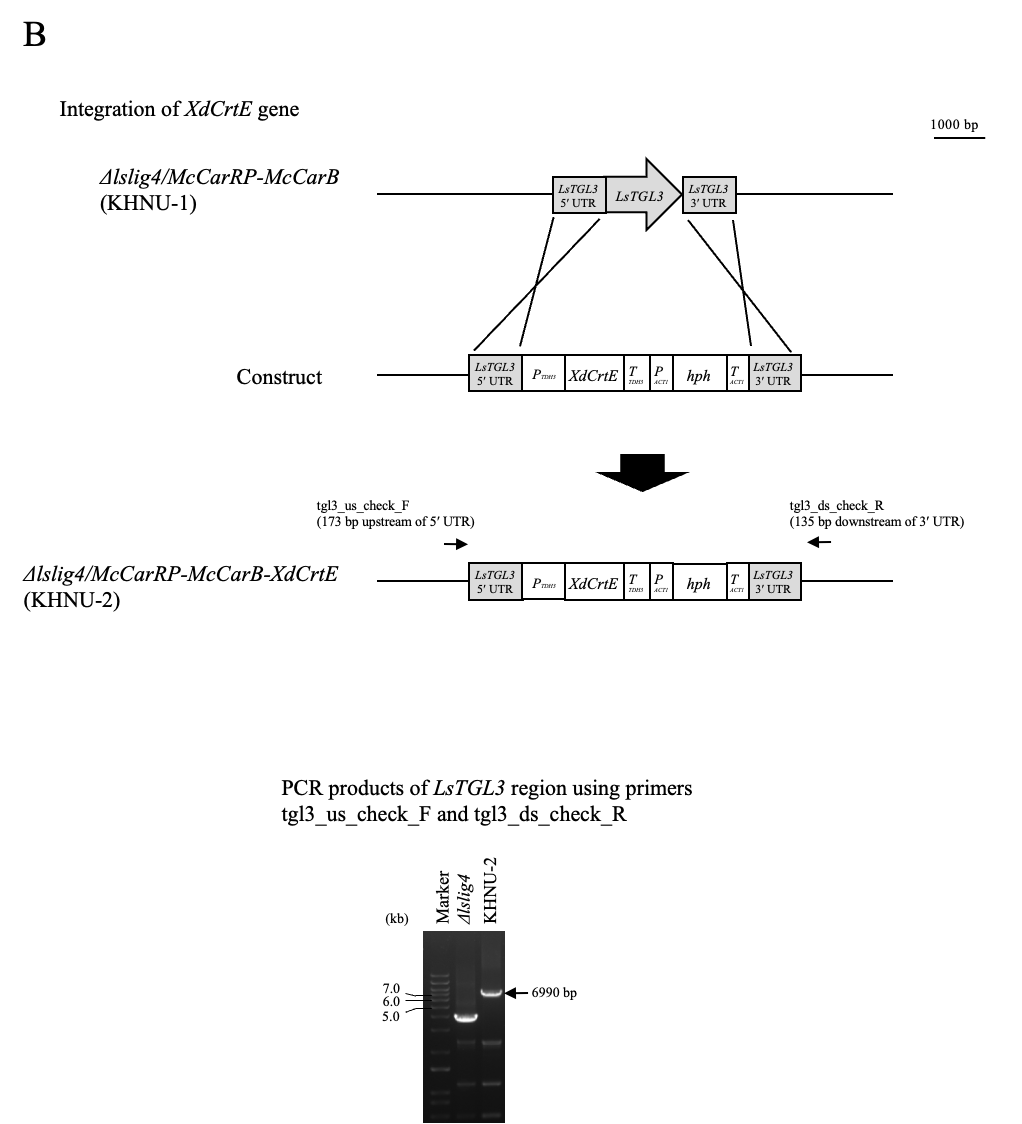
**


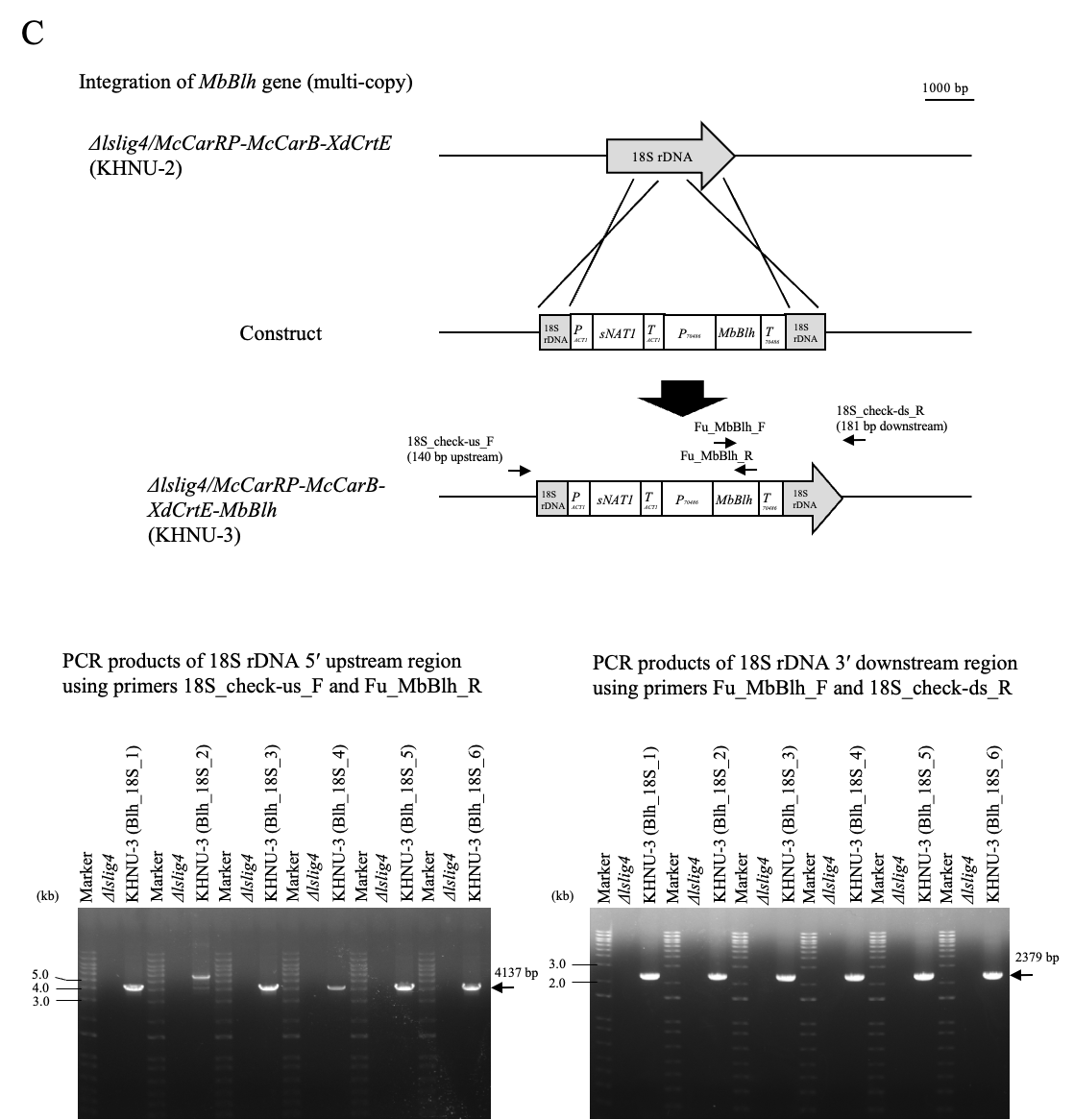


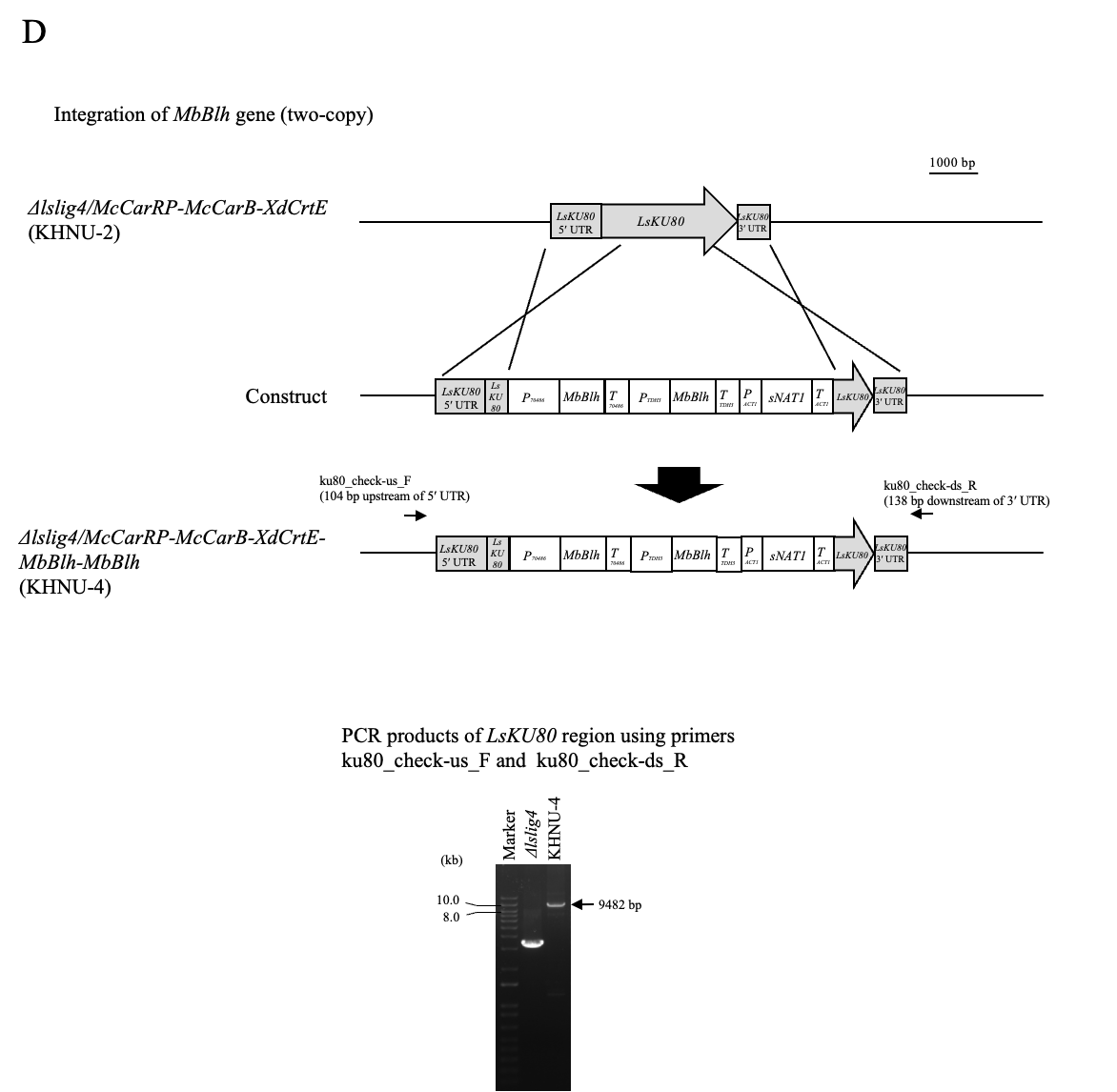


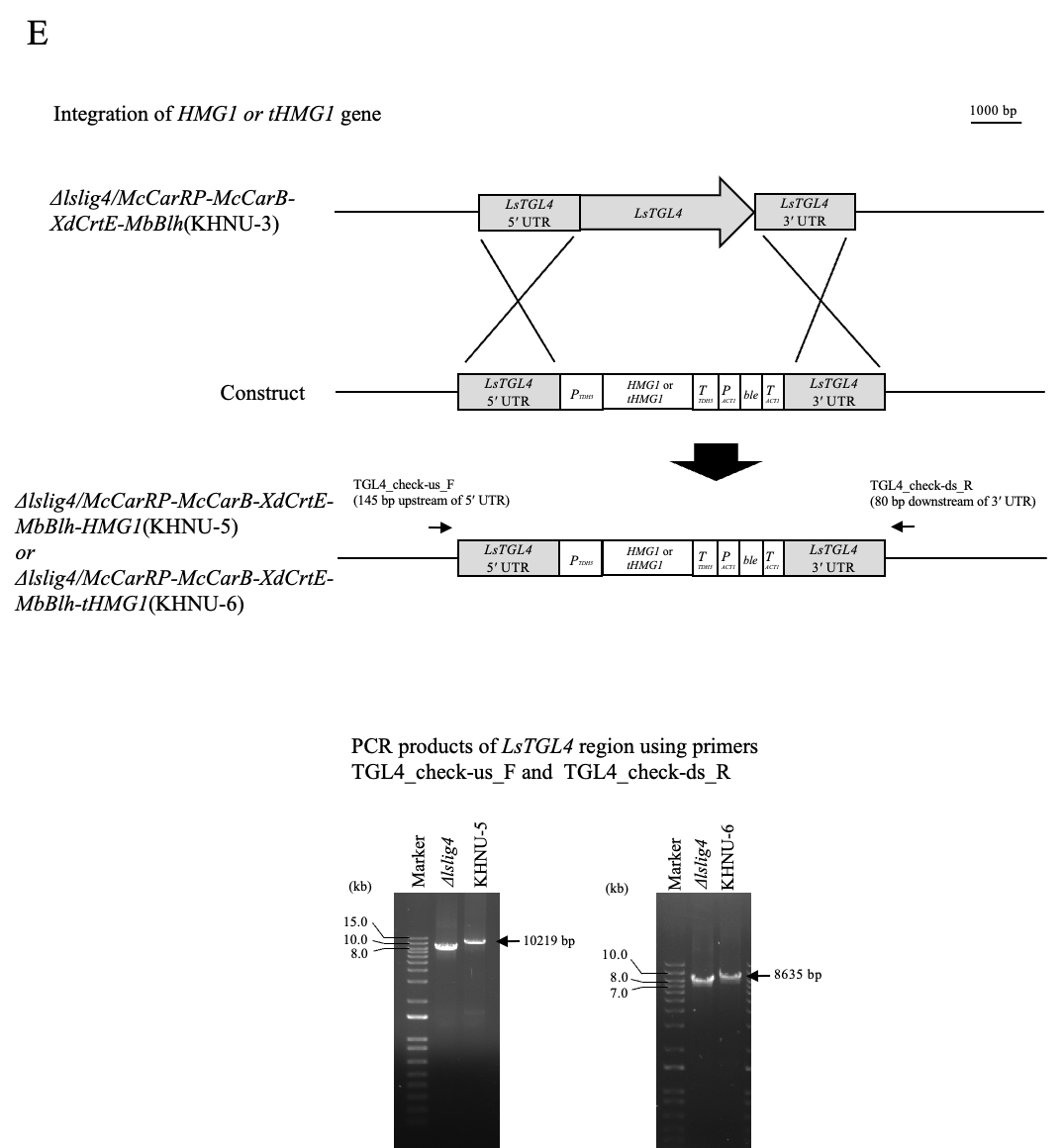


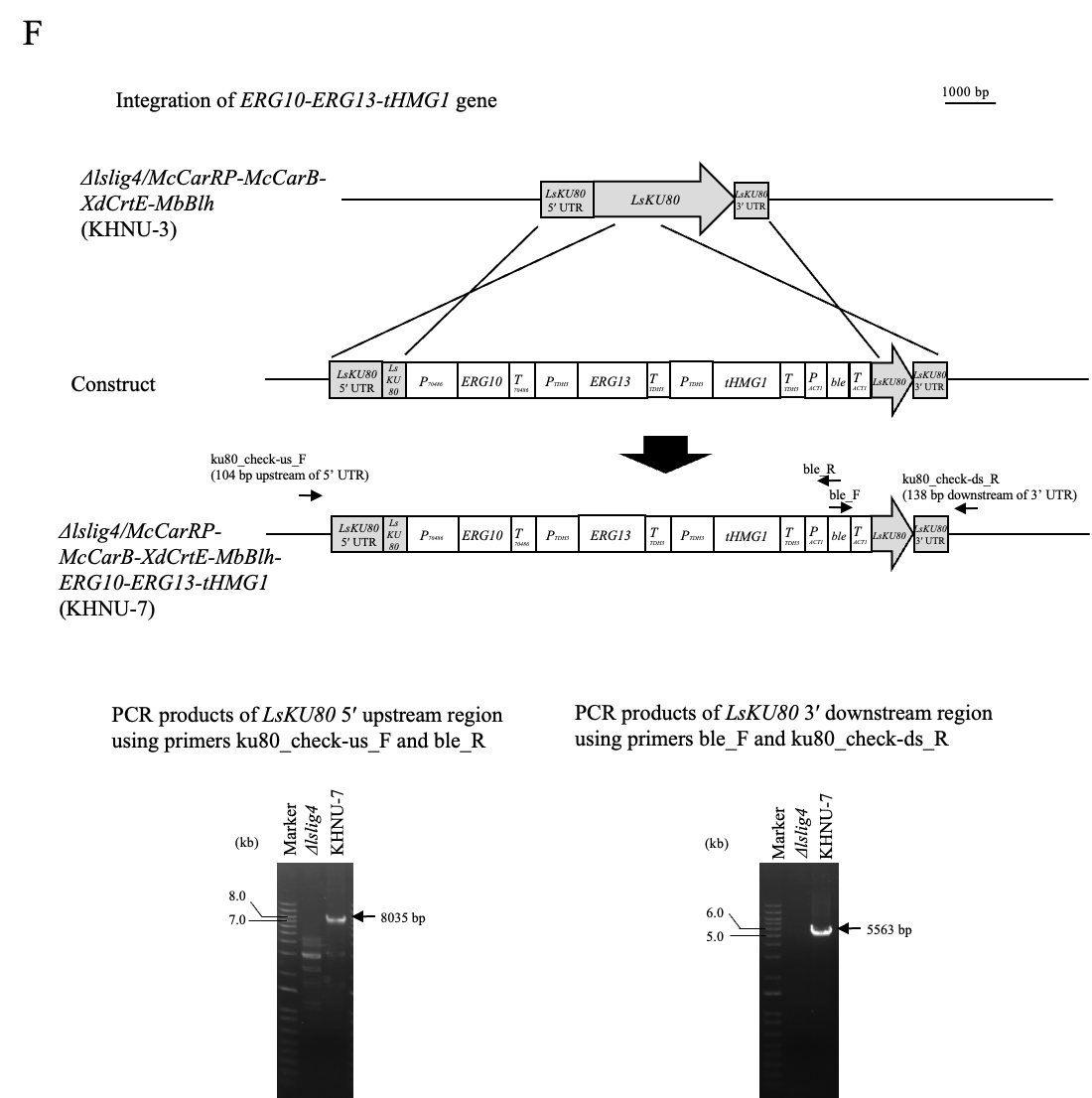


**Figure S1 Genetic engineering strategy and confirmation of gene integration by PCR**

A) Integration strategy of the *McCarRP* and *McCarB* overexpression cassette into the *LsLIG4* locus and verification of integration by PCR. PCR was performed using a MiniAmp Plus Thermal Cycler (Thermo Fisher Scientific). PCR was performed using genomic DNA as a template and the primer sets lig4_us_check_F/KanR_R or KanR_F/lig4_ds_check_R under the following conditions: 95°C for 2 min (initial denaturation), followed by 35 cycles of 95°C for 10 s, 64°C for 30 s, and 68°C for 30 s/kb. Arrows in the schematic diagram indicate the primers used for PCR analysis. Detection of the correct-sized PCR products from both the 5′ upstream region (11,047 bp) and the 3′ downstream region (4,463 bp) of *LsLIG4* confirms the correct recombination event and integration.

B) Integration strategy of the *XdCrtE* overexpression cassette into the *LsTGL3* locus and verification of integration by PCR. Confirmation of the gene integration for the target gene was performed as described in panel (A). The primer set used for PCR analysis was tgl3_us_check_F /tgl3_ds_check_R. The correct recombination event in the *LsTGL3* locus results in a 6,990 bp PCR product.

C) Integration strategy of the *MbBlh* overexpression cassette into the 18S rDNA locus and verification of integration by PCR. Confirmation of the gene integration for the target gene was performed as described in panel (A). The primer sets used for PCR analysis were 18S_check-us_F/ Fu_MbBlh_R and Fu_MbBlh_F/18S_check-ds_R. Detection of the correct-sized PCR products from both the 5′ upstream region (4,137 bp) and the 3′ downstream region (2,379 bp) of 18S rDNA confirms the correct recombination event and integration.

D) Integration strategy of the *MbBlh* overexpression cassette into the *LsKU80* locus and verification of integration by PCR. Confirmation of the gene integration for the target gene was performed as described in panel (A). The primer set used for PCR analysis was ku80_check-us_F /ku80_check-ds_R. The correct recombination event in the *LsKU80* locus results in a 9,482 bp PCR product.

E) Integration strategy of the *HMG1* or *tHMG1* overexpression cassette into the *LsTGL4* locus and verification of integration by PCR. Confirmation of the gene integration for the target gene was performed as described in panel (A). The primer set used for PCR analysis was TGL4_check-us_F /TGL4_check-ds_R. The correct recombination event for *HMG1* integration results in a 10,219 bp PCR product, and the correct recombination event for *tHMG1* integration results in an 8,635 bp PCR product.

F) Integration strategy of the *ERG10, ERG13* and *tHMG1* overexpression cassette into the *LsKU80* locus and verification of integration by PCR. Confirmation of the gene integration for the target gene was performed as described in panel (A). The primer sets used for PCR analysis were ku80_check-us_F/ble_R and ble_F/ku80_check-ds_R. Detection of the correct-sized PCR products from both the 5′ upstream region (8,035 bp) and the 3′ downstream region (5,563 bp) of *LsKU80* confirms the correct recombination event and integration.


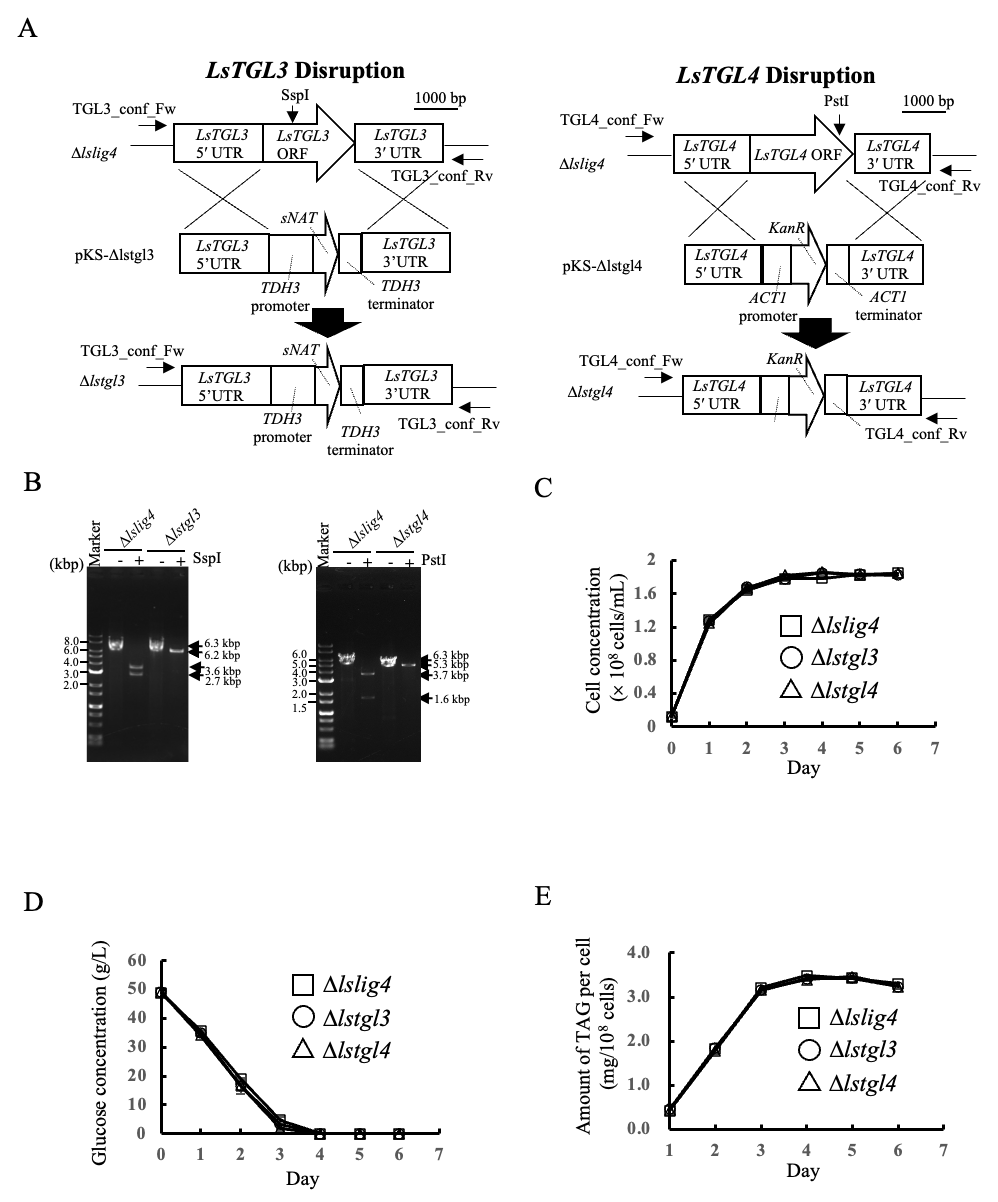


**Figure S2 Disruption of the *LsTGL3* and *LsTGL4* genes and phenotypic characterization of the ∆*lstgl3* and ∆*lstgl4* strains**

A) Strategies for *LsTGL3* and *LsTGL4* gene disruption. Detailed constructions of the disruption plasmids pKS-∆lstgl3 and pKS-∆lstgl4 are described in the Supplementary Methods.

B) Verification of *LsTGL3* and *LsTGL4* disruptions by colony PCR. Colonies were used as templates for amplification with the primer sets TGL3_conf_Fw (5′-CAGAACTGGATGCTGCTGCTG-3′)/TGL3_conf_Rv (5′-CGCAGTTCAAGGAGTACTTGG-3′) or TGL4_conf_Fw (5′-GTCAGCACTTGAGCTTCTTCATGC-3′)/TGL4_conf_Rv (5′-CGTGCAAAAGAGCCTCAGCC-3′). The PCR products were digested with SspI or PstI and analyzed by agarose gel electrophoresis. SspI sites are present in the *LsTGL3* locus but absent in the s*NAT* cassette; thus, 3.6- and 2.7-kbp fragments were observed in the ∆*lslig4* strain, while a single 6.2-kbp fragment was detected in the ∆*lstgl3* strain. PstI sites are present in the *LsTGL4* locus but absent in the *KanR* cassette; thus, 1.6- and 3.7-kbp fragments were observed in the ∆*lslig4* strain, while a single 5.3-kbp fragment was detected in the ∆*lstgl4* strain.

C–E) Comparison of cell concentration (cells/mL), glucose concentration (g/L), and amount of TAG per cell (mg/10^8^ cells) between ∆*lslig4*, ∆*lstgl3,* and ∆*lstgl4* strains. The data are indicated as the mean ± SEM of the three independent experiments.


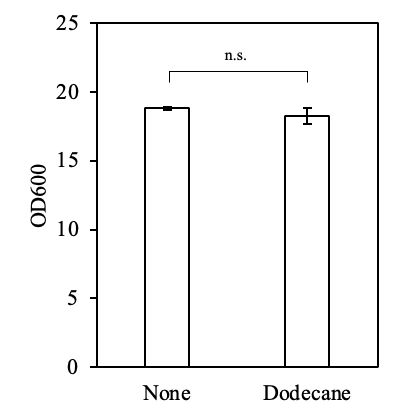


**Figure S3 Growth of the vitamin A-producing strain KHNU-3 in culture supplemented with dodecane**

KHNU-3 was precultured overnight in YPD medium at 30°C, 300 rpm. The preculture (2%) was then inoculated into 4 mL of fresh YPD medium with or without 20% (v/v) dodecane containing 1% (w/v) BHT in large test tubes and cultured for 72 hours. OD600 was measured at 72 hours post-inoculation using a UV-1900i spectrophotometer (Shimadzu). (Student’s t test; n.s., not statistically significant).


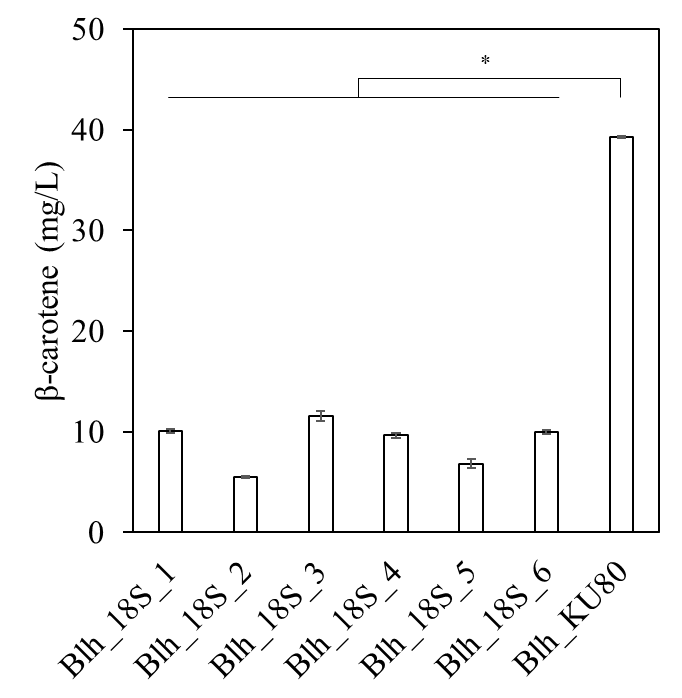


**Figure S4 β-carotene accumulation in vitamin A-producing strains harboring multi-copy *MbBlh***

KHNU-3 (Blh_18S_1-6) and KHNU-4 (Blh_KU80) were precultured 2 days in YPD medium at 30°C, 300 rpm. The precultures (2%) were then inoculated into 4 mL of fresh YPD medium in large test tubes and cultured for 96 hours. At the mid-log phase, 800 μL of dodecane with 1% (w/v) BHT was added. Vitamin A in the dodecane layer and β-carotene in the cells were quantified using HPLC (LC-20AD, Shimadzu). Statistically significant differences in β-carotene titers between KHNU-3 and KHNU-4 are indicated by an asterisk. Data are presented as the mean ± SEM determined from three independent experiments. (Dunnett’s test; **P*<0.05)

**A**

**
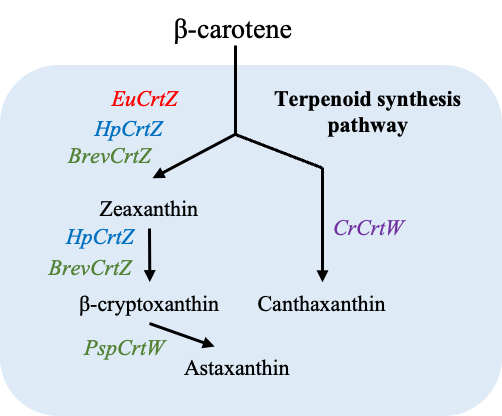
**

**B**

**
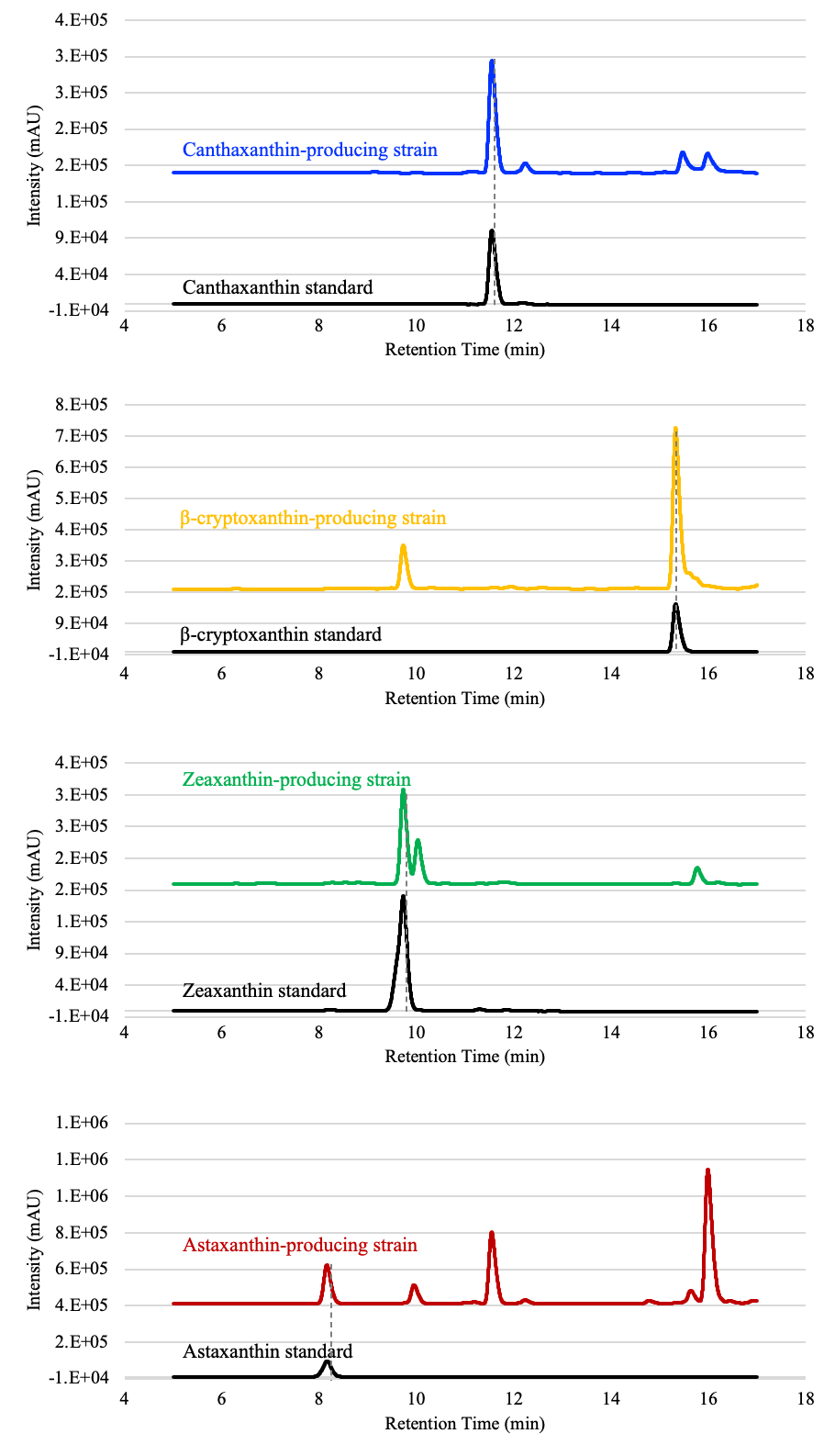
**

**C**

**
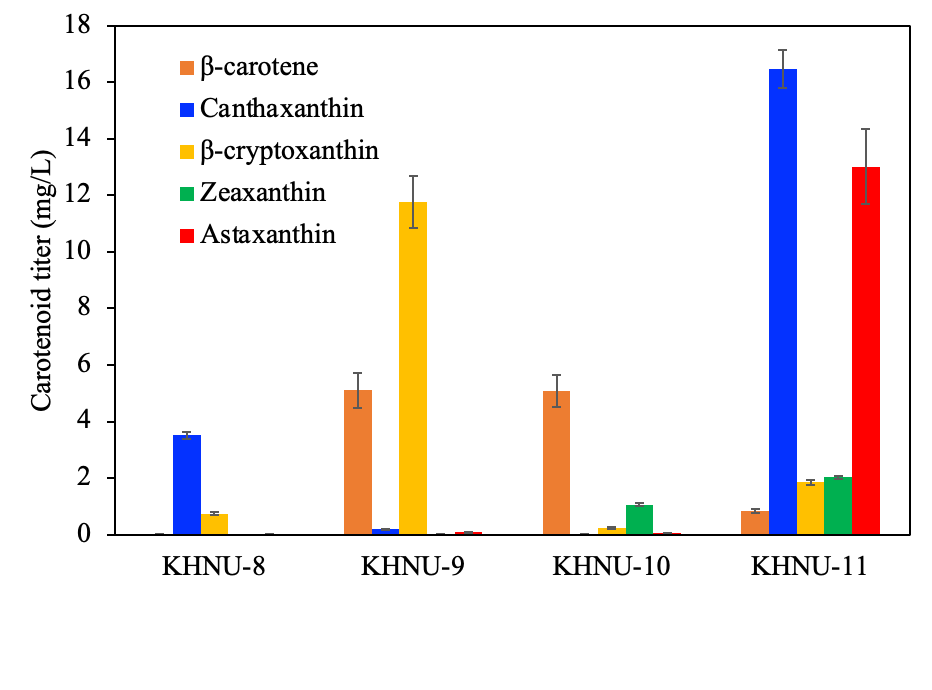
**

**Figure S5 Biosynthesis of canthaxanthin, β-cryptoxanthin, zeaxanthin, and astaxanthin in *L. starkeyi*.**

(A) Schematic representation of the heterologous terpenoid synthesis pathway introduced into β-carotene–producing *L. starkeyi* strains. Expression of β-carotene hydroxylases (*CrtZ*) from *Erwinia uredovora* (*EuCrtZ*), *Haematococcus pluvialis* (*HpCrtZ*), and *Brevundimonas* sp. SD212 (*BrevCrtZ*) leads to the formation of zeaxanthin and β-cryptoxanthin, while β-carotene ketolases (*CrtW*) from *Chlamydomonas reinhardtii* (*CrCrtW*) and *Paracoccus* sp. N81106 (*PspCrtW*) catalyze the conversion of β-carotene and its derivatives to canthaxanthin and astaxanthin, respectively. Arrows indicate enzymatic steps catalyzed by the respective heterologous enzymes.

(B) *L. starkeyi* transformants expressing heterologous carotenoid-modifying enzymes were precultured in YPD medium at 30°C and 300 rpm for 2 days. Subsequently, 2% (v/v) of the preculture was inoculated into 4 mL of fresh S medium (0.5% (NH₄)₂SO₄, 0.1% KH₂PO₄, 0.01% NaCl, 0.3% yeast extract, 0.05% MgSO₄·7H₂O, 0.01% CaCl₂·2H₂O, and 2% glucose, pH 5.5) in large test tubes and cultured for 96 h. Cells were disrupted using a Multi-Beads Shocker (Yasui Kikai) at 2500 rpm for 15 min at 4 °C. The cell lysates were centrifuged, and the resulting supernatants were analyzed using an HPLC system (LC-20AD, Shimadzu). Carotenoids were detected at 450 nm.

(C) Quantitative analysis of carotenoid production in the engineered strains corresponding to the chromatograms shown in (B). The quantitative measurements were performed in an independent cultivation experiment under the same culture conditions. Data represent the mean ± standard deviation from three independent biological replicates (n = 3).

**Supplementary Methods**

**Construction of ∆*lstgl3*, ∆*lslig4*, and ∆*lslig3*∆*lslig4* strains**

For the construction of pKS-∆lstgl3, *L. starkeyi* genomic DNA was used as a template to amplify the 5′ and 3′ untranslated regions (UTRs) of *LsTGL3* by PCR with the primer sets TGL3 5′UTR_fwd (AAAAGCTGGGTACCGGGCCCCCAACTGCCCTCGTCTCTC)/TGL3 5′UTR_rev (AGCAAATTAAGTGGCGACCAATGCGGATC) and TGL3 3′UTR_fwd (CCCGCTACATCAGCTGTCACGAATTTCGACAATTAC)/TGL3 3′UTR_rev (TCGACCTCGAGGGGGGGCCCTCAACACCCTGAATACCAGAGG). The *sNAT1* expression cassette was amplified from pKS-sNAT [2] using the primers TGL3 NAT_fwd (TGGTCGCCACTTAATTTGCTGAAGCGGTTTGCC)/TGL3 NAT_rev (GTGACAGCTGATGTAGCGGGTGGTGATG), and the vector backbone was amplified from pBluescript KS (+) using vector_fwd (GGGCCCCCCCTCGAGGTCGACGGTATC)/vector_rev (GGATCCGGGCCCGGTACCCAGCTTTTG). The four DNA fragments were assembled using the NEBuilder HiFi DNA Assembly Master Mix (New England Biolabs) to construct pKS-∆lstgl3.

For the construction of pKS-∆lstgl4, *L. starkeyi* genomic DNA was used as a template to amplify the 5′ and 3′ UTRs of *LsTGL4* by PCR with primer sets TGL4 5′UTR_fwd (TGGGTACCGGGCCCGGATCCCCACAGGTATCTTCTTCCTTGC)/TGL4 5′UTR_rev (TAGACGGTAACGCTGCTGATACTAACCCC) and TGL4 3′UTR_fwd (CTATACTCCTATCAGTAGTACGACGGGGTC)/TGL4 3′UTR_rev (TCGACCTCGAGGGGGGGCCCCCAGATAGACCCTTGGCATAC). The *KanR* expression cassette was amplified from pKS-KanR [2] using primers TGL4 KanR_fwd (ATCAGCAGCGTTACCGTCTACCGCTGACG)/TGL4 KanR_rev (TACTACTGATAGGAGTATAGAGTTGAATTTAATGGACGTTG), and the vector backbone was amplified from pBluescript KS (+) with primers vector_fwd (GGGCCCCCCCTCGAGGTCGACGGTATC)/vector_rev (GGATCCGGGCCCGGTACCCAGCTTTTG). The four resulting fragments were assembled using the NEBuilder HiFi DNA Assembly Master Mix (New England Biolabs) to generate pKS-∆lstgl4.

For strain construction, NotI-digested DNA fragments of pKS-∆lstgl3 and pKS-∆lstgl4 were transformed into the *L. starkeyi* *∆lslig4* strain to obtain the ∆*lstgl3* and ∆*lstgl4* mutants, respectively. Furthermore, NotI-digested pKS-∆lstgl4 was transformed into the ∆*lstgl3* strain to generate the double-deletion strain ∆*lstgl3* ∆*lstgl4*.

**Construction of canthaxanthin-producing strain**

To construct the canthaxanthin-producing *L. starkeyi* strain KHNU-8, the β-carotene ketolase (*CrtW*) derived from *Chlamydomonas reinhardtii* was amplified from synthetic DNA using the primer pairs 18S insertion CrCrtW 5′UTR_fwd (5′-TATTCAACTTTCACAATGGGACCAGGCATTCA-3′)/18S insertion CrCrtW 5′UTR_rev (5′-GCGAGACGACGTCTACGCCATAACGCCGACCGGAG-3′). The vector backbone was amplified from pKS/18S/MbBlh using the primer pairs MbBlh_fwd (5′-TAGACGTCGTCTCGCTTCCTCTCGAACCCGCACTATC-3′)/MbBlh_rev (5′-TGTGAAAGTTGAATATAGATAGTAAGAGCTTTTTCGAAGAG-3′). The amplified fragments were assembled using the In-Fusion Snap Assembly Master Mix (Takara Bio). The resulting plasmid was linearized with ApaI and transformed into *L. starkeyi* KHNU-2, generating the canthaxanthin-producing strain KHNU-8.

**Construction of β-cryptoxanthin-producing strain**

To construct the β-cryptoxanthin-producing *L. starkeyi* strain KHNU-9, the β-carotene hydroxylase (*CrtZ*) derived from *Haematococcus pluvialis* was amplified from synthetic DNA using the primer pairs 18S insertion HpCrtZ _fwd (5′-TATTCAACTTTCACAATGTTGTCCAAGTTGCA-3′)/18S insertion HpCrtZ _rev (5′-GCGAGACGACGTCTACCTTTTGCTCCAATCAAGTTCCAA-3′). The vector backbone was amplified from pKS/18S/MbBlh using the primer pairs MbBlh_fwd/MbBlh_rev. The resulting PCR fragments were assembled using the In-Fusion Snap Assembly Master Mix (Takara Bio). The assembled plasmid was linearized with ApaI and introduced into *L. starkeyi* KHNU-2 via transformation, generating the β-cryptoxanthin-producing strain KHNU-9.

**Construction of zeaxanthin-producing strain**

To construct the zeaxanthin-producing *L. starkeyi* strain KHNU-10, the β-carotene hydroxylase (*CrtZ*) derived from *Erwinia uredovora* was amplified from synthetic DNA using the primer pairs 18S insertion EuCrtZ 5′UTR_fwd (5′-TATTCAACTTTCACAATGTTGTGGATTTGGAA-3′)/18S insertion EuCrtZ 5′UTR_rev (5′-GCGAGACGACGTCTACTTTCCAGAGGCAGGTTCGTC-3′). The vector backbone was amplified from pKS/18S/MbBlh using the primer pairs MbBlh_fwd/MbBlh_rev. The resulting PCR fragments were assembled using the In-Fusion Snap Assembly Master Mix (Takara Bio). The resulting plasmid was linearized with ApaI and transformed into *L. starkeyi* KHNU-2, generating the zeaxanthin-producing strain KHNU-10.

**Construction of astaxanthin-producing strain**

To construct the astaxanthin-producing *L. starkeyi* strain KHNU-11, the β-carotene hydroxylase (*CrtZ*) derived from *Brevundimonas* sp. SD212 was amplified from synthetic DNA using the primer pairs 18S insertion BrevCrtZ 5′UTR_fwd (5′-TATTCAACTTTCACAATGGCGTGGTTGACCTG-3′)/18S insertion BrevCrtZ 5′UTR_rev (5′-GCGAGACGACGTCTAAGCACCACTACTGCTAGAGCC-3′). The vector backbone was amplified from pKS/18S/MbBlh using the primer pairs MbBlh_fwd/MbBlh_rev. The PCR fragments were assembled using the In-Fusion Snap Assembly Master Mix (Takara Bio). The resulting plasmid is pKS/18S/BrevCrtZ.

The β-carotene ketolase gene (*CrtW*) from *Paracoccus* sp. N81106 was amplified from synthetic DNA using the primer pair pKS insertion PspCrtW 5′UTR_fwd (5′-ATTACCACCACAACAATGTCAGCGCATGCTCT-3′) and pKS insertion PspCrtW 5′UTR_rev (5′-ACAAAAAAACGTCTAGGCAGTGTCGCCTTTAGTTCTC-3′). The vector backbone was amplified from pKS-LsLIG4-P70486-McCarRP(n)-P3813-McCarB(n)-P63951-MbBlh-P3900-ScEnv9-KanR-ApaI, which was constructed as described below, using the primer pair pKS_fwd (5′-TAGACGTTTTTTTGTGTTATTGCGCGCTTGTTTTGGAAC-3′) and pKS_rev (5′-TGTTGTGGTGGTAATTAACAGATTAAGACTATTGTTGCGG-3′). The resulting PCR fragments were assembled using the In-Fusion Snap Assembly Master Mix.

The expression cassette P3900-PspCrtW-T3900 was amplified from the assembled plasmid using the primer pair pt3900_fwd (5′-CACCCTGCTCTTCCCTCAAAACCTAGACAGCGGCTCTA-3′) and pt3900_rev (5′-TCCCTTGAGCTAGTTGATAAAGTACAACAATCAATGACTC-3′). The vector backbone was again amplified from pKS/18S/BrevCrtZ using MbBlh_WF (5′-AACTAGCTCAAGGGACGTGCTATTC-3′) and MbBlh_RF (5′-GGGAAGAGCAGGGTGGGCTTG-3′), and both PCR fragments were assembled using the In-Fusion Snap Assembly Master Mix, resulting in the *BrevCrtZ*-*PspCrtW* co-expression plasmid. The resulting plasmid was linearized with ApaI and transformed into *L. starkeyi* KHNU-2, generating the astaxanthin-producing strain KHNU-11.

**Construction of plasmid pKS-LsLIG4-P70486-McCarRP(n)-P3813-McCarB(n)-P63951-MbBlh-P3900-ScEnv9-KanR-ApaI**

To construct the plasmid pKS-LsLIG4-P70486-McCarRP(n)-P3813-McCarB(n)-P63951-MbBlh-P3900-ScEnv9-KanR-ApaI, *L. starkeyi* CBS1807 genomic DNA was used as a template to amplify the 5′- and 3′-UTRs of *LsLIG4*, the promoter and terminator regions of transcript ID 70486 (for *McCarRP* expression), transcript ID 3813 (for *McCarB* expression), *TEF1* (for *MbBlh* expression), and transcript ID 3900 (for *ScENV9* expression).

PCR amplification was performed using the following primer sets (5′→3′):

LsLIG4-5′-Fw: ataagcttgatgggcccgcggccgcCTCAGACTTTACCACAGATACGG/LsLIG4-5′-Rv: gagtagagcgagAGTTACCACAATTATATGCACATGGAGTC

70486p-(McCarRP)n-Fw: aattgtggtaactCTCGCTCTACTCTACTCTAGTCTAAC/70486p-(McCarRP)n-Rv: aagtgagaagcatTGTGAAAGTTGAATATAGATAGTAAGAGCTTTTTCG

70486t-(McCarRP)n-Fw: aacactatctaaACGTCGTCTCGCTTCCTCTC/70486t-(McCarRP)n-Rv: accaccagatctGGGAAGAGCAGGGTGGGC

3813p-(McCarB)n-Fw: ccctgctcttcccAGATCTGGTGGTCCCGCC/3813p-(McCarB)n-Rv: cttcttagacatGATGCTTTAATGTGATGTAGACGAAATGCAAAAAG

3813t-(McCarB)n-Fw: aacgtcatctaaACGGCATGAACAAGGTACTTAG/3813t-(McCarB)n-Rv: aatcttaatggtACAAGTAAAACAACTGCGAGTAGAC

TEF1p-(MbBlh)-Fw: taatctttcaaaATGGGCCTTATGCTGATTGATTG/TEF1p-(MbBlh)-Rv: gcacgaaaaccttTTAGTTTTTGATCTTGATCCGACTAGAATGTG

TEF1t-(MbBlh)-Fw: atcaaaaactaaAAGGTTTTCGTGCTCTTGTTTGTC/TEF1t-(MbBlh)-Rv: tctaggttttgaAGGGATCAGTATCAGACTCCG

3900p-(ScENV9)-Fw: gatactgatccctTCAAAACCTAGACAGCGGC/3900p-(ScENV9)-Rv: tgggatctaacatTGTTGTGGTGGTAATTAACAGATTAAGACTATTG

3900t-(ScENV9)-Fw: tttgatatataaACGTTTTTTTGTGTTATTGCGCG/3900t-(ScENV9)-Rv: ggtagacggtaaGATAAAGTACAACAATCAATGACTCTTGCAC

LsLIG4-3′-Fw: actctatactcctGCCTGTTATAGAAGTCAAGTTCGC/LsLIG4-3′-Rv: gggctgcaggaattcgatggggcccCACATGCACATCTTTATCGAGGAC

The codon-optimized *McCarRP(n)* and *McCarB(n)* genes for *L. starkeyi* were synthesized by GenScript Japan Inc. (Tokyo, Japan) and amplified using the following primers:

McCarRP(n)-Fw: tcaactttcacaATGCTTCTCACTTACATGGAGGTTC/McCarRP(n)-Rv: gcgagacgacgtTTAGATAGTGTTGAGGTTACGCATCTTAC

McCarB(n)-Fw: acattaaagcatcATGTCTAAGAAGCACATCGTTATTATCGG/McCarB(n)-Rv: ttgttcatgccgtTTAGATGACGTTAGAGTTGTGGACAC

The *MbBlh* gene was amplified from pKS/18S/MbBlh using the primer pair:

MbBlh-Fw: taatctttcaaaATGGGCCTTATGCTGATTGATTG/MbBlh-Rv: gcacgaaaaccttTTAGTTTTTGATCTTGATCCGACTAGAATGTG

The codon-optimized *ScENV9* gene for *L. starkeyi* was synthesized by Eurofins Genomics and amplified using the primer pair:

ScENV9-Fw: accaccacaacaATGTTAGATCCCAGAATCCTTCCC/ScENV9-Rv: acaaaaaaacgtTTATATATCAAAGCCTCTATCGCGAAGC

The *ACT1* promoter–*KanR*–*ACT1* terminator cassette was amplified from pKS/lig4/McCarRP-McCarB using the primer pair:

ACT1t-KanR-ACT1t-Fw: gttgtactttatcTTACCGTCTACCGCTGACG/ACT1t-KanR-ACT1t-Rv: tctataacaggcAGGAGTATAGAGTTGAATTTAATGGACGTTG

The vector backbone was amplified from pKS-18S-hph using:

Vector-Fw: GGGCCCCATCGAATTCCTGCAGCC/Vector-Rv: GCGGCCGCGGGCCCATCA

All amplified DNA fragments were assembled using the NEBuilder HiFi DNA Assembly Master Mix (New England Biolabs), yielding the final plasmid pKS-LsLIG4-P70486-McCarRP(n)-P3813-McCarB(n)-P63951-MbBlh-P3900-ScEnv9-KanR-ApaI.

**Supplementary References**

1. Madeira F, Madhusoodanan N, Lee J, Eusebi A, Niewielska A, Tivey ARN, et al. The EMBL-EBI Job Dispatcher sequence analysis tools framework in 2024. Nucleic Acids Research. 2024;52**:**W521–W525.

2. Oguro Y, Yamazaki H, Ara S, Shida Y, Ogasawara W, Takagi M, et al. Efficient gene targeting in non-homologous end-joining-deficient *Lipomyces starkeyi* strains. Curr Genet. 2017;63**:**751–763.
